# The symmetry spectrum in a hybridising, tropical group of rhododendrons

**DOI:** 10.1111/nph.18083

**Published:** 2022-03-29

**Authors:** Valerie L. Soza, Ricardo Kriebel, Elizabeth Ramage, Benjamin D. Hall, Alex D. Twyford

**Affiliations:** ^1^ 7284 Department of Biology University of Washington Seattle WA 98115 USA; ^2^ 5228 Department of Botany University of Wisconsin‐Madison Madison WI 53706 USA; ^3^ Institute of Evolutionary Biology School of Biological Sciences University of Edinburgh Charlotte Auerbach Road Edinburgh EH9 3FL UK; ^4^ Royal Botanic Garden Edinburgh 20A Inverleith Row Edinburgh EH3 5LR UK

**Keywords:** flower symmetry, introgression, morphometrics, New Guinea, phylogenetics, *Rhododendron*, Southeast Asia, Vireyas

## Abstract

- Many diverse plant clades possess bilaterally symmetrical flowers and specialised pollination syndromes, suggesting that these traits may promote diversification. We examined the evolution of diverse floral morphologies in a species‐rich tropical radiation of *Rhododendron*.
- We used restriction‐site associated DNA sequencing on 114 taxa from *Rhododendron* sect. *Schistanthe* to reconstruct phylogenetic relationships and examine hybridisation. We then captured and quantified floral variation using geometric morphometric analyses, which we interpreted in a phylogenetic context.
- We uncovered phylogenetic conflict and uncertainty caused by introgression within and between clades. Morphometric analyses revealed flower symmetry to be a morphological continuum without clear transitions between radial and bilateral symmetry. Tropical *Rhododendron* species that began diversifying into New Guinea *c*. 6 million years ago expanded into novel floral morphological space.
- Our results showed that the evolution of tropical *Rhododendron* is characterised by recent speciation, recurrent hybridisation and the origin of floral novelty. Floral variation evolved via changes to multiple components of the corolla that are only recognised in geometric morphometrics with both front and side views of flowers.

## Introduction

The specialisation of flowers is a key factor underlying the evolutionary success and ecological dominance of angiosperms. Flower symmetry, in particular bilateral symmetry in which a flower exhibits one plane of symmetry along the dorsoventral axis, is believed to facilitate pollinator attraction, visitation, efficiency, precision and specialisation (reviewed in Neal *et al*., 1998). Bilateral flower symmetry promotes more specific interactions with pollinators and more precise pollen placement (Sargent, 2004), leading to increased diversification rates (O’Meara *et al*., 2016) compared with radial symmetry. As such, understanding transitions in flower symmetry and the evolution of novel floral morphologies is essential for answering long‐standing questions about the origin and diversity of species richness in angiosperms.

Tropical plant radiations present the richest botanical diversity and exhibit diversity in floral form to match. From the largest flower on Earth, *Rafflesia arnoldii*, to the marked specialised floral structures of the sunbird‐pollinated bird of paradise, *Strelitzia reginae*, tropical plants demonstrate an astonishing range of floral forms, including many forms found nowhere else (Endress, 1996). Pinpointing the evolutionary drivers of diverse tropical floral morphologies is at the heart of understanding angiosperm diversity, but many factors impede this work. Explosive species radiations are typically characterised by phylogenetic complexity, in which shallow species divergences, potentially associated with hybridisation, obscure inferences of evolutionary relationships (Schley *et al*., 2020). At a more fundamental level, a lack of baseline taxonomic knowledge impedes evolutionary research in many tropical groups (Lagomarsino & Frost, 2020). Furthermore, the extensive morphological diversity present in these groups can be difficult to analyse and classify and rarely fits simple models of floral shape that were developed for model temperate species.

Geometric morphometrics has been used to quantify floral shape variation in macroevolutionary studies (Wilson *et al*., 2017; Smith & Kriebel, 2018; Kriebel *et al*., 2020; Reich *et al*., 2020) but has been rarely used to quantify flower symmetry variation, only in microevolutionary studies (Gómez *et al*., 2006; Savriama *et al*., 2012; Hsu *et al*., 2015; Wang *et al*., 2015; Berger *et al*., 2017). These microevolutionary studies used landmark data to detect changes and characterise variation in floral symmetry in population‐level and mutant analyses. Rarely has this method been used to characterise flower symmetry variation across clades (Gardner *et al*., 2016). In addition, most of these studies, with the exception of Hsu *et al*. (2015) and Wang *et al*. (2015), examined symmetry from only the front view of the flower. Geometric morphometrics could be useful for quantifying floral symmetry variation in diverse groups, for which it is difficult to qualitatively describe the flowers as radially or bilaterally symmetric, by using both front and side views of flowers. This approach, in conjunction with phylogenomic analyses, could be used to understand floral diversification in tropical plant groups and the mode and tempo of evolution of novel floral forms.

**Fig. 1:**
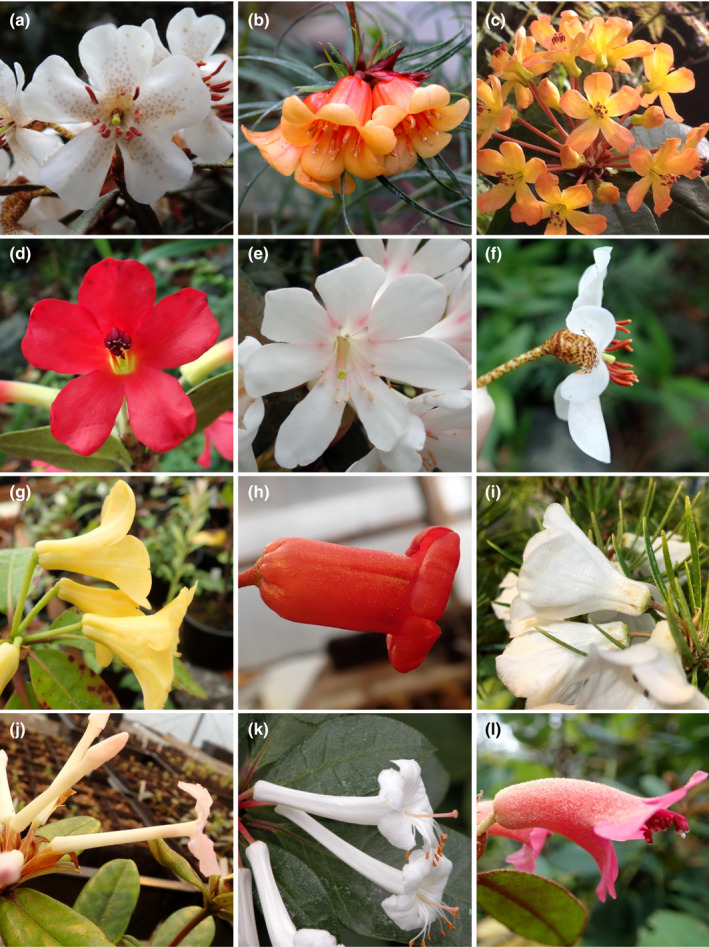
Flower symmetry variation in *Rhododendron* sect. *Schistanthe*. (a–e) Front views of flowers. (f–l) Side views of flowers. (a) *R. himantodes* (subsect. *Malayovireya*) with weakly bilaterally symmetric flowers. (b) *R. stenophyllum* (subsect. *Euvireya*) with radially symmetric flowers. (c) *R. macgregoriae* (subsect. *Euvireya*) with bilaterally symmetric flowers. (d) *R. christi* (subsect. *Euvireya*) with bilaterally symmetric flowers. (e) *R. konori* (subsect. *Euvireya*) with deviation from pentamerous flowers and bilaterally symmetric flowers. (f) *R. himantodes* (subsect. *Malayovireya*) with short corolla tubes. (g) *R. robinsonii* (subsect. *Euvireya*) with oblique corolla tubes. (h) *R. pauciflorum* (subsect. *Euvireya*) with straight corolla tube. (i) *R. taxifolium* (subsect. *Euvireya*) with short, straight corolla tubes. (j) *R. edanoi* subsp. *pneumonanthum* (subsect. *Euvireya*) with straight, long corolla tubes. (k) *R. cruttwellii* (subsect. *Euvireya*) with long corolla tubes curved upwards. (l) *R. beyerinckianum* (subsect. *Euvireya*) with long corolla tubes curved downwards.

The genus *Rhododendron* (Ericaceae) is a large group of over 1000 species, with notable species richness in the tropics. This diversity arose via southward dispersal from the group’s origin in Northeast Asia, which is likely to have been facilitated by mountain building in the Malay Archipelago (Shrestha *et al*., 2018), followed by an eastward migration to New Guinea and Australia (Goetsch *et al*., 2011; Webb & Ree, 2012; Landis *et al*., 2013). Malesian *Rhododendron* species are classified in sect. *Schistanthe* (Craven *et al*., 2011), commonly known as vireyas, and comprise over 300 species that have been reported as diploid (Janaki Ammal *et al*., 1950; Jones & Brighton, 1972; Atkinson *et al*., 2000) and show the widest range of floral morphology in the genus (Argent, 2015) (Fig. 1). The section is unusual for a diverse tropical group in having been subject to extensive taxonomic research and having a recent taxonomic account covering all described species (Argent, 2015).

*Rhododendron* is considered one of the five most species‐rich vascular plant genera in New Guinea (Cámara‐Leret *et al*., 2020), comprising 171 species (Argent, 2015). Prior phylogenetic studies in sect. *Schistanthe* using chloroplast or several nuclear genes found that traditional subsectional classifications (Sleumer, 1966) based on flower shape and leaf scales do not reflect the group’s evolutionary history (Brown, 2003; Brown *et al*., 2006a,b; Goetsch *et al*., 2011). Instead, clades reflect geographical distributions across Southeast Asia, exhibiting a general west to east dispersal pattern and subsequent dramatic diversification in New Guinea, which was confirmed by Xing & Ree (2017). However, relationships within these geographic clades remain largely unresolved (Brown *et al*., 2006a,b), and monophyly of sect. *Schistanthe* and its subsections was not supported (Goetsch *et al*., 2011). This phylogenetic uncertainty has largely been attributed to recent species divergence, coupled with hybridisation between sympatric taxa.

Extensive variation in *Rhododendron* flower symmetry is achieved through various means, such as corolla pigment patterns and structural variation in the corolla, stamens and style (Fig. 1) (Stevens *et al*., 2004). Corollas in sect. *Schistanthe* are reported to be radially or bilaterally symmetric (Argent *et al*., 2007; Craven *et al*., 2011). However, corolla symmetry has not been designated in species descriptions for many taxa and is often difficult to distinguish due to variation within the group. Despite this, a radially symmetric corolla has been reconstructed as the ancestral state for the group based on front views of flowers and limited sampling (Berry *et al*., 2018). Corolla tubes, however, vary in sect. *Schistanthe* from straight to curved adaxially (downwards) or curved abaxially (upwards) (Fig. 1f–l) (Stevens, 1976). The only taxa denoted as bilaterally symmetric in taxonomic treatments are those that exhibit adaxially curved corolla tubes (Fig. 1l) (Sleumer, 1966; van Royen & Kores, 1982; Argent, 2015). However, based on Stevens’s (1976) description of flower types within sect. *Schistanthe*, we predict the origin of at least two different types of bilateral symmetry in the group based on curvature of the corolla tube (adaxial or abaxial; Fig. 1k,l).

The goals of our study were to investigate the phylogenetic complexity and floral diversification, with regard to flower symmetry, of sect. *Schistanthe*. Specifically, we aimed to (1) resolve the phylogeny of *Rhododendron* sect. *Schistanthe* using genome‐wide data, (2) investigate whether hybridisation underlies phylogenetic complexity, and (3) characterise flower symmetry variation and evolution in this species radiation. We used restriction‐site associated DNA sequencing (RAD‐seq) to resolve the phylogenetic history of this group and found evidence for introgression within and between clades. Our curve‐based approach to morphometrics of corolla symmetry demonstrated that bilateral symmetry in sect. *Schistanthe* is a continuous characteristic that is made up of multiple corolla components. We identified corolla symmetry types in the section and inferred at least two transitions to novel bilateral symmetry within the New Guinean radiation.

## Materials and Methods

### Sampling and sequencing

For phylogenomic analyses, we collected 147 samples representing 114 taxa from *Rhododendron* sect. *Schistanthe* and 17 outgroups (Kron, 1997; Kron *et al*., 2002; Gillespie & Kron, 2010; Schwery *et al*., 2015; Shrestha *et al*., 2018) (Supporting Information Table S1). Plant samples were primarily from cultivated accessions at the Rhododendron Species Botanical Garden, Federal Way, WA, USA and the Royal Botanic Garden Edinburgh, UK. Fresh or silica gel‐dried leaf tissue was collected for genomic DNA extraction using the DNeasy^®^ Plant Mini Kit (Qiagen^®^, Valencia, CA, USA) according to a modified protocol (Soza *et al*., 2019).

We followed the method of Etter *et al*. (2011) for creating RAD‐seq libraries using the *Pst*I restriction enzyme. We prepared a total of six RAD‐seq library pools, each containing 12–33 taxa, to provide suitable sequencing coverage per sample. We used 400 ng of starting DNA for each sample and reduced all reaction components proportionally. Pooled libraries were sequenced individually using 100‐bp paired‐end reads on a HiSeq 2500 system (Illumina Inc., San Diego, CA, USA) by the QB3 Vincent J. Coates Genomics Sequencing Laboratory, University of California, Berkeley, USA, or the Genomics and Cell Characterization Core Facility, University of Oregon, Eugene, USA. Further details of the molecular methods are given in Methods S1.

### Identification of RAD loci

We demultiplexed RAD‐seq data from each library using process_radtags in Stacks v.1.47 (Catchen *et al*., 2011, 2013) under default settings, discarding reads with low quality scores, rescuing barcodes and RAD tags, and filtering for adapters by allowing two mismatches in adapter sequences. We then removed polymerase chain reaction (PCR) duplicates from our data using the paired‐end reads and clone_filter in Stacks. We used Ipyrad v.0.7.17 (Eaton, 2014; Eaton & Overcast, 2020) to identify loci within and across samples for phylogenetic reconstructions. We determined the optimal clustering threshold of 0.91 empirically (Fig. S1; Methods S2) and used the *Rhododendron delavayi* Franch. genome (Zhang *et al*., 2017) as the reference. Based on our RAD optimisation results (Fig. S2a–c; Methods S2), we allowed a maximum of eight indels, 34 single nucleotide polymorphisms (SNPs) and a shared heterozygosity value of 0.3 per locus to obtain alignments for phylogenetic inference. We generated a total of four datasets that allowed different amounts of sample coverage across loci: requiring a minimum of 4, 37, 74 and 111 samples per locus (Fig. S2d; Table S2). We refer to these datasets subsequently as min4, min37, min74 and min111, respectively.

### Phylogenetic reconstructions and estimates of introgression

We reconstructed phylogenetic relationships across sect. *Schistanthe* and outgroups using a maximum likelihood (ML) topology search in RAxML v.8.2.11 (Stamatakis, 2014) and the multispecies coalescent model in SVDQuartets (Chifman & Kubatko, 2014, 2015) for each of the four datasets above. Rooting for phylogenetic trees followed relationships from prior studies (Kron, 1997; Gillespie & Kron, 2010; Schwery *et al*., 2015; Rose *et al*., 2018; Shrestha *et al*., 2018). Topological support was estimated using bootstrap (bs) support and Quartet Sampling v.1.3.1 (Pease *et al*., 2018). Further details of the phylogenetic methods are given in Methods S3.

We then used the *D*‐statistic (ABBA‐BABA test) (Green *et al*., 2010; Durand *et al*., 2011) as implemented in Dsuite v.0.3r21 (Malinsky *et al*., 2021) to assess the extent of hybridisation across the group. Three different methods were used (Methods S4) and corresponding *P*‐values were adjusted with the Benjamini–Hochberg correction using rstatix (Kassambara, 2020) in R v.4.0.3 (R Core Team, 2020) for generating a heatmap.

### Molecular dating and biogeography

Due to the large size of our molecular datasets, we obtained estimates of clade ages for sect. *Schistanthe* using penalised likelihood (Sanderson, 2002) in treePL (Smith & O’Meara, 2012). We used the ML topology from the min4 dataset to optimise parameters in treePL and as a topological constraint to generate 1000 rapid bootstrap replicates with branch lengths, under the GTRCAT model in RAxML v.8.2.12, for confidence intervals, as described in Maurin (2020). We used four fossil calibrations that have been used in prior studies of Ericaceae (Schwery *et al*., 2015; Xing & Ree, 2017) to provide minimum ages for the crowns of *Erica*, *Kalmia* and *Rhododendron* and a maximum age for the root. Further details of the molecular dating are given in Methods S5. The resulting chronogram was visualised in R v.4.0.3 using treeio v.1.12.0 (Wang *et al*., 2020) and ggtree v.2.2.4 (Yu, 2020).

While the lack of species‐level resolution and presence of phylogenetic conflict prevented formal fine‐scale biogeographic and diversification analyses, we estimated ages of clades and highlighted clades’ geographic distributions based on extant distributions. We classified geographic areas in the archipelago into five regions based on important faunal boundaries: west of Huxley’s Line, the Philippines, Sulawesi and the Lesser Sunda Islands, the Moluccas and east of Lydekker’s Line. We used geographic distributions from Argent (2015) for taxa sampled in our study to estimate clade occurrence in each geographic region. We used ggplot2, rnaturalearth (South, 2017) and sf (Pebesma, 2018) in R v.4.0.3 to generate a map of these geographic regions.

### Morphometric analyses

To quantify the symmetry of flowers from species in sect. *Schistanthe*, we used the geometric morphometric technique of elliptical Fourier analysis (eFa) (Kuhl & Giardina, 1982; Kincaid & Schneider, 1983; Claude, 2008), which is used to extract quantitative information from curved structures, such as corolla tubes and corolla lobes in *Rhododendron*. In addition, we used this approach to characterise the complex variation in floral symmetry present in this clade from front and side views of flowers. eFa was implemented with the R library momocs (Bonhomme *et al*., 2014). The inputs for eFa were two‐dimensional black outlines placed on white backgrounds and saved as jpg files. To this end, we took macro photographs of the front and side views of flowers from cultivated material placed against a plain background, or obtained images from the literature for our sampled taxa (Table S1), and used Gimp v.2.10.8 (The GIMP Development Team, 2018) to create and save the outlines (Methods S6). To align the outlines and avoid their inadvertent twisting during eFa, three and four landmarks were placed on the side‐ and front‐view outlines, respectively. In side‐view outlines, one landmark was placed at the base of the corolla tube, a second at the end of the upper lip and a third at the end of the lower lip. For front‐view outlines, one landmark was placed on each side at the middle, one at the top and one at the bottom. After placing the landmarks, a full generalised procrustes alignment between outlines of each structure was conducted and the resulting aligned outlines were evaluated to determine the number of harmonics needed to achieve enough harmonic power for the eFa. The resulting eFa coefficients were used to calculate a mean shape per species and then summarised using principal component analysis (PCA). As we were interested in overall variation among species and not between the left and right side of the front view of the corolla, within‐specimen variation between opposing halves was removed with the *rm_sym* function in momocs before PCA.

To characterise floral symmetry, we used *K*‐means clustering with the R library nbclust (Charrad *et al*., 2014) and tested for the presence of distinct morphological groups using the first three principal components (PC) for each view of the corollas. nbclust provides 30 indices to determine the best number of clusters in the data and proposes a best clustering scheme based on the majority rule. The possible numbers of clusters were set to the minimum allowed of two and a maximum of 20. We grouped taxa in morphospaces by *K*‐means clustering, clade and corolla colour (Methods S6). Clades were assigned based on the SVDQuartets analyses of the min4 dataset.

Last, to examine the evolution of floral morphology during the evolutionary history of sect. *Schistanthe*, we tested for the presence of evolutionary shifts using an Ornstein–Uhlenbeck (OU) process modelling approach implemented in the R library l1ou (Khabbazian *et al*., 2016). The OU process is useful in the context of flower evolution because it models natural selection directly (Butler & King, 2004) and flowers are usually under selective pressure by pollinators. Its implementation with l1ou allows for the detection of significant shifts on a phylogeny into multiple morphological regimes without having to *a priori* determine where changes might have occurred. We ran two multivariate analyses, one for the front and one for the side view of flowers. In each analysis, the first three PCs were included. To choose the best shift configuration, we used the phylogenetic Bayesian information criterion (pBIC), which has been shown to be conservative and therefore reduce the possibility of detecting unsupported shifts.

## Results

### Phylogenomic conflict highlights introgression within *Rhododendron* sect. *Schistanthe*

**Fig. 2:**
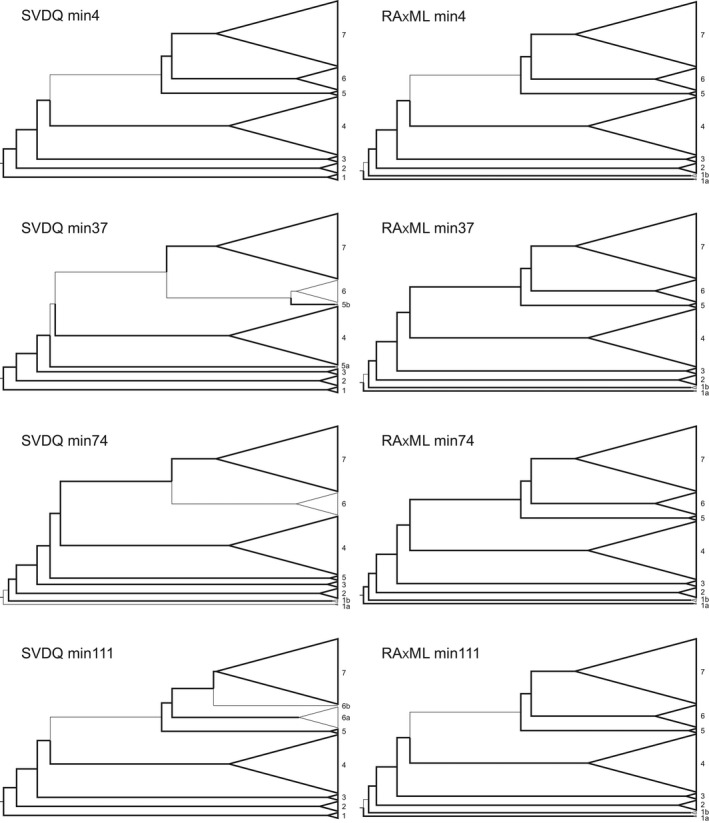
Comparison of topologies obtained from four different datasets using two phylogenetic reconstruction methods, SVDQuartets (SVDQ) and RAxML. The four different datasets (min4, min37, min74 and min111) represent different amounts of sample coverage across loci, requiring a minimum of 4, 37, 74 and 111 samples per locus. Topologies show a summary of relationships among seven main clades (1–7) in *Rhododendron* sect. *Schistanthe*. Branches with bootstrap (bs) support ≥ 70% or 90% are indicated by thicker branches for SVDQ or RAxML topologies, respectively. When clades are supported with bs values ≥ 70% or 90% for SVDQ or RAxML topologies, respectively, they are indicated with thicker triangles.

**Fig. 3:**
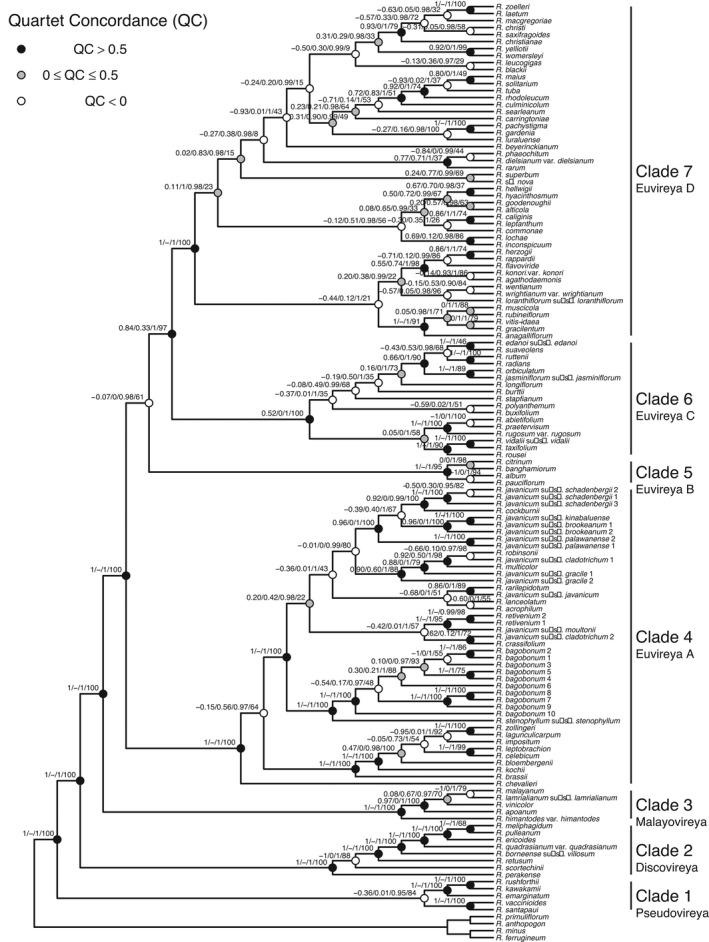
SVDQuartets consensus topology of *Rhododendron* sect. *Schistante* that was generated from the min4 dataset, which required four samples per locus. This analysis used 117 121 unlinked single nucleotide polymorphisms (SNPs). Support values at nodes for the ingroup are shown in the following order: Quartet Concordance/Quartet Differential/Quartet Informativeness/bootstrap. Seven main clades (1–7) were identified and referred to based on subsectional classification: Pseudovireya, Discovireya, Malayovireya, Euvireya A, Euvireya B, Euvireya C and Euvireya D, respectively.

We estimated phylogenetic relationships across 147 geographically widespread samples from *Rhododendron* sect. *Schistanthe* and outgroups using four RAD‐seq datasets with different levels of missing data and two reconstruction methods: ML in RAxML and the multispecies coalescent model in SVDQuartets (SVDQ). All RAxML and SVDQ reconstructions supported the monophyly of sect. *Schistanthe* (Figs 2, 3, S3–S9). However, these results also highlighted phylogenetic conflict and uncertainty with respect to seven clades. For example, comparing across these two phylogenetic methods and four datasets, we found strong bs support (100% across all SVDQ and RAxML topologies) for four clades (clades 2, 3, 4 and 7) but conflict in three clades (clades 1, 5 and 6) (Figs 2, 3, S3–S9).

We then used bs support to identify the most strongly supported topology from each reconstruction method and Quartet Sampling to verify relationships and/or confirm conflict. In both RAxML and SVDQ reconstructions, the min4 dataset resulted in the most strongly supported topology based on bs values (Figs 3, S3). We re‐examined support for clades in the RAxML and SVDQ topologies from this dataset using Quartet Concordance (QC) and Quartet Differential (QD) scores. In the RAxML topology, seven clades were strongly supported (QC = 1, QD = N/A): 1a, 1b, 2–5 and 7 (Fig. S3). In the SVDQ topology, six clades were strongly supported (QC = 1, QD = N/A): 2–5 and 7 (Fig. 3). Based on relationships across these topologies and using the classification of Craven *et al*. (2011), we recognised seven main clades in sect. *Schistanthe*. Clade 1, 2 and 3 corresponded to subsects. *Pseudovireya*, *Discovireya* and *Malayovireya*, respectively, while clades 4–7 corresponded to subsect. *Euvireya*. Therefore, we referred to clades 1–7 as Pseudovireya, Discovireya, Malayovireya, Euvireya A, Euvireya B, Euvireya C and Euvireya D, respectively (Fig. 3).

**Fig. 4:**
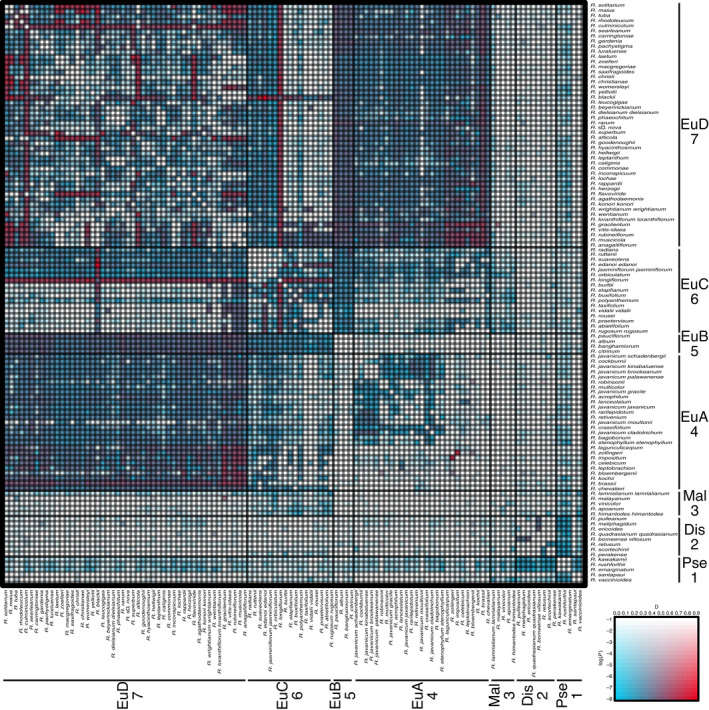
Heatmap of *D*_BBAA_‐statistics in *Rhododendron* sect. *Schistanthe*. *D*‐statistics were calculated by inferring species relationships based on the frequency of BBAA patterns. P2 and P3 taxa are arranged along the vertical and horizontal axes. Clade designations and numbers are indicated along taxon names: Dis, Discovireya; EuA, Euvireya A; EuB, Euvireya B; EuC, Euvireya C; EuD, Euvireya D; Mal, Malayovireya; and Pse, Pseudovireya. The most significant *D*‐statistic found for two species across all possible P1 taxa is indicated by heatmap cells. Red cells indicate higher *D*‐statistics (presence of introgression), blue cells indicate lower *D*‐statistics (absence of introgression), increasing colour saturation indicates greater significance based on *P*‐values and white cells indicate lack of information.

Comparing the two most strongly supported topologies from each reconstruction method, we found that conflict between topologies may be due in part to introgression as 41% (RAxML) and 46% (SVDQ) of nodes had QD scores < 0.5 (Figs 3, S3). We then assessed the history of hybridisation across sect. *Schistanthe* using the *D*‐statistic (ABBA‐BABA test) as implemented in Dsuite. Based on three methods used to calculate *D*‐statistics, we found differing amounts of introgression, and here we highlight our results from *D*_BBAA_ as moderate estimates of the *D*‐statistic (Figs 4, S10, S11). We observed significantly high *D*‐statistics within clades Euvireya A (clade 4) (e.g. *R. impositum* and *R. zollingeri*, *D*_BBAA_ = 0.7, *P* < 1 × 10^–15^), Euvireya C (clade 6) (e.g. *R. longiflorum* and *R. polyanthemum*, *D*_BBAA_ = 0.65, *P* < 1 × 10^–15^) and Euvireya D (clade 7) (e.g. *R. blackii* and *R. maius*, *D*_BBAA_ = 0.76, *P* < 1 × 10^–15^). We found strong support for introgression between clades Euvireya A (clade 4) and Euvireya D (clade 7) (e.g. *R. brassii* and *R. gracilentum*, *D*_BBAA_ = 0.61, *P* < 1 × 10^–15^) and between clades Euvireya C (clade 6) and Euvireya D (clade 7) (e.g. *R. blackii* and *R. edanoi*, *D*_BBAA_ = 0.86, *P* < 1 × 10^–15^). We did not find strong signals of introgression with Pseudovireya (clade 1), Discovireya (clade 2), Malayovireya (clade 3) and Euvireya B (clade 5) but also lacked enough information to confidently determine this in these groups (Fig. 4). Overall, the evidence from *D*‐statistics pointed to introgression within and between clades within sect. *Schistanthe* that is probably causing topological conflict.

### Temporal origins of clades in *Rhododendron* sect. *Schistanthe*

**Fig. 5:**
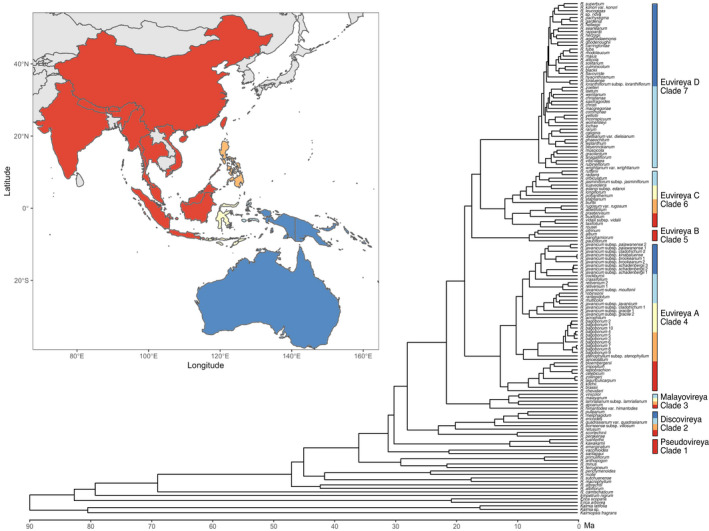
Chronogram of *Rhododendron* sect. *Schistanthe* and outgroups. Penalised likelihood and four fossil calibrations were used with the RAxML topology from the min4 dataset to infer ages (Ma, million years ago). Extant geographic distribution of sect. *Schistanthe* is indicated by coloured regions on the map, which are based on important faunal boundaries in the Malay Archipelago: red, west of Huxley’s Line; orange, the Philippines; yellow, Sulawesi and the Lesser Sunda Islands; light blue, the Moluccas; and dark blue, east of Lydekker’s Line. Clade occurrence in each geographic region is indicated by coloured vertical bars.

We subsequently estimated ages for clades within sect. *Schistanthe* to provide a temporal context for downstream morphological analyses of floral variation using the RAxML min4 topology and four fossil calibrations with penalised likelihood in treePL. Our analyses estimated a mean (95% confidence interval) crown age of 31.04 (30.66–31.38) million years ago (Ma) for sect. *Schistanthe* (Fig. 5). *Rhododendron* subsect. *Euvireya* had a mean crown age of 18.48 (18.25–18.69) Ma; its subclades, as represented by clades Euvireya A–D (clades 4–7), had mean crown ages of 12.65 (12.50–12.80), 7.89 (7.76–8.02), 11.89 (11.74–12.01) and 6.33 (6.25–6.41) Ma, respectively (Fig. 5). Based on current species distributions, we mapped geographic regions to clades and estimated that the New Guinean radiation, as evidenced by the diversity present in clade Euvireya D (clade 7), began *c*. 6.33 Ma (Fig. 5). All taxa sampled in this clade occur east of Lydekker’s Line except for *R. zoelleri*, which occurs in the Moluccas as well.

### Characterising flower symmetry variation using both front and side views of corollas

**Fig. 6:**
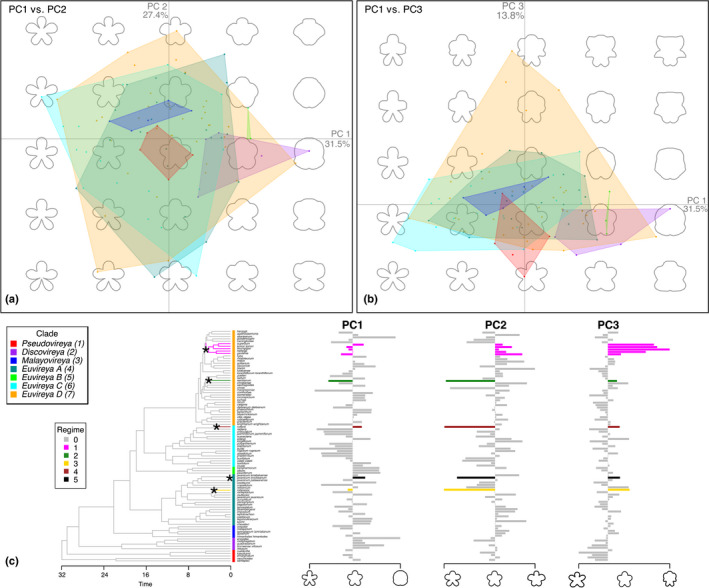
Morphospace variation of the front view of corollas from *Rhododendron* sect. *Schistanthe*. (a, b) Coloured dots and polygons correspond to clade designations in (c). Polygons represent total morphospace occupied by each clade. (c) Result of multivariate shift detection analysis under an Ornstein–Uhlenbeck process with l1ou. The best shift configuration included five shifts (each indicated with an asterisk) from the background regime (grey) into five different regimes (coloured edges). Example outlines corresponding to the mean and extreme principal component (PC) values are shown below the bar plot of tip data for each PC axis. Tips are labelled with square symbols coloured based on clade designations in (c).

**Fig. 7:**
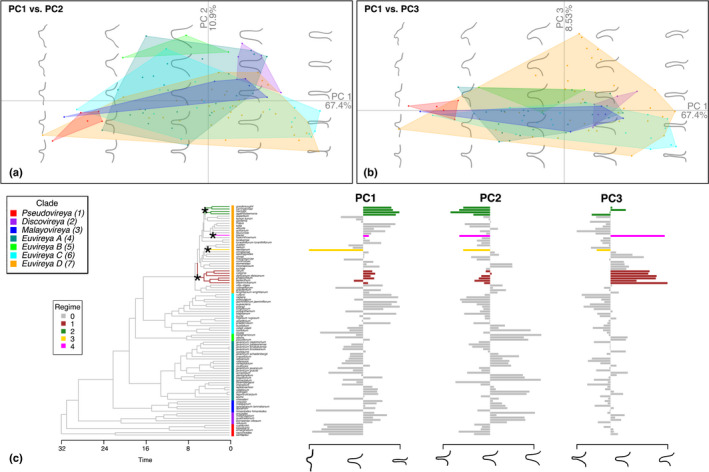
Morphospace variation of the side view of corollas from *Rhododendron* sect. *Schistanthe*. (a, b) Coloured dots and polygons correspond to clade designations in (c). Polygons represent total morphospace occupied by each clade. (c) Result of multivariate shift detection analysis under an Ornstein–Uhlenbeck process with l1ou. The best shift configuration included four shifts (each indicated with an asterisk) from the background regime (grey) into four different regimes (coloured edges). Example outlines corresponding to the mean and extreme principal component (PC) values are shown below the bar plot of tip data for each PC axis. Tips are labelled with square symbols coloured based on clade designations in (c).

To characterise flower symmetry variation in sect. *Schistanthe*, we used eFa and PCA to quantify and summarise the overall symmetry of front and side views of corollas from taxa sampled in our study. We generated a total of 168 outlines representing 95 taxa for the front view of the corolla (taxon mean *n* = 1.77 (SD = 0.83)) and 166 outlines representing 97 taxa for the side view of the corolla (taxon mean *n* = 1.71 (SD = 0.91)) (Fig. S12). The outlines, both raw and landmarked, are available in Dryad (please refer to Data availability). For both corolla views, > 99% of harmonic power was achieved with 32 harmonics, so this number was used in the eFa. The PCA of the eFa coefficients from the front of the corolla resulted in a total of 73% of the variation explained by the first three PCs (PC1 = 32%, PC2 = 27% and PC3 = 14%) (Fig. 6a,b). PC1 explained variation in depth of petal lobing. PC2 captured variation in asymmetry between the upper and lower parts of the flower. PC3 represented variation in the number and shape of lateral petal lobes as well as differences in the angle and depth of lobing between petal lobes in the upper vs lower parts of the flower. The PCA of the side of the corolla explained 87% of the variation in the first three PCs (PC1 = 67%, PC2 = 11% and PC3 = 9%) (Fig. 7a,b). PC1 explained variation in corolla tube length. PC2 explained variation in corolla tube width and asymmetry in reflexing of the upper vs lower petal lobes. PC3 explained the curvature of corolla tube, tube angle and length differences between the upper and lower lobes.

In both views, an apparent morphological continuum made it difficult to identify transitions between radial and bilateral symmetry. Therefore, we used *K*‐means clustering to test for distinct symmetry phenotypes in sect. *Schistanthe*. After evaluating 30 indices for the optimal number of clusters, nbclust used the majority rule to identify five clusters in the front view and two clusters in the side view (Fig. S13). The clusters from the front view appeared to delineate symmetry types: three clusters of bilaterally symmetric flowers and two clusters of more radially symmetric flowers, with each cluster differing in petal number, lobing, curvature, angle and/or length (Fig. S13a,b). However, in side view, *K*‐means clustering did not identify distinct bilaterally symmetric phenotypes, but rather it identified clusters that were mainly defined by the length of the tubes (Fig. S13c,d). All flowers appeared bilaterally symmetric in side view due to asymmetry between the upper and lower lobes of the corolla (Fig. S13c,d). Despite the morphological continuum and different components of corolla symmetry, we identified five distinct symmetry phenotypes in sect. *Schistanthe* using the front views of corollas.

### Characterising flower symmetry evolution in a morphological radiation

We examined clade occupancy of corolla morphospace as a means to understand flower symmetry evolution within sect. *Schistanthe* given phylogenetic conflict. When considering the front‐view morphospace between PC1 and PC2, clades Pseudovireya (clade 1), Discovireya (clade 2), Malayovireya (clade 3) and Euvireya B (clade 5) occupied relatively small areas of the morphospace and exhibited radially symmetric flowers (Fig. 6a). By contrast, clades Euvireya A (clade 4), Euvireya C (clade 6) and Euvireya D (clade 7) occupied the largest morphological spaces exhibiting different types of radially and bilaterally symmetric flowers (Fig. 6a). When PC3 was included, we observed clade Euvireya D (clade 7) expanding into a new morphological space where corollas became more than five‐lobed due to two extra lobes in lateral positions, and upper petals had wider angles between the lobes and/or shallower lobes compared with the lower petals (Fig. 6b), generating a novel form of bilateral symmetry. Taken together, clade Euvireya D (clade 7) explored more morphospace than other clades when considering the majority of variation from all three PCs representing the front views of corollas.

We then investigated shifts in floral evolution in clades through time using the RAxML min4 topology. Across all PCs for the front view of corollas, one shift was detected in clade Euvireya D (clade 7) that was shared by multiple species (*R. gardenia*, *R. hellwigii*, *R. konori*, *R. leucogigas* and *R. superbum*) (Fig. 6c). This shift confirmed the novel origin of bilaterally symmetric corollas due to increased lateral lobes and upper petals with wider angles between the lobes and/or shallower lobes than the lower petals in clade Euvireya D (clade 7) (Fig. 6c).

As PC1 represented depth of petal lobing but not symmetry, we used the origin of clades in PC2 and PC3 to examine symmetry evolution. Using the origin of clades in PC2, early diverging clades Pseudovireya (clade 1), Discovireya (clade 2) and Malayovireya (clade 3) appeared to have ancestral phenotypes of radially symmetric flowers (Fig. 6c). However, when PC3 was included, clades Pseudovireya (clade 1) and Discovireya (clade 2) showed origins near a phenotype that had bilaterally symmetric flowers due to wider angles between lower petal lobes than between upper petal lobes (Fig. 6c). From this ancestral phenotype, transitions to radial symmetry and other types of bilateral symmetry occurred within clades (Fig. 6c). Therefore, sect. *Schistanthe* may have been ancestrally bilaterally symmetric with wider angles between the lower petal lobes than between the upper lobes. Transitions to radial symmetry and other types of bilateral symmetry occurred in the group with a novel type of bilateral symmetry arising in clade Euvireya D (clade 7).

We also examined clade occupancy of side‐view corolla morphospace as a means to understand flower symmetry evolution. When examining variation in PC1 and PC2, clades Pseudovireya (clade 1), Discovireya (clade 2) and Euvireya B (clade 5) occupied the smallest morphological spaces, followed by clade Malayovireya (clade 3), while clades Euvireya A (clade 4), Euvireya C (clade 6) and Euvireya D (clade 7) occupied the largest morphological spaces (Fig. 7a). When PC3 was included, we observed clade Euvireya D (clade 7) expanding into a new morphological space where corolla tubes become curved with longer upper lobes than lower lobes (Fig. 7b), generating a novel form of bilateral symmetry. Taken together, clade Euvireya D (clade 7) explored more morphospace than other clades when considering the majority of variation from all three PCs representing the side views of corollas.

We again investigated shifts in floral evolution in clades through time, taking into account the side view of corollas. Across all PCs, two additional shifts were detected in clade Euvireya D (clade 7) that were shared by multiple species (Fig. 7c). One shift was shared by *R. agathodaemonis*, *R. carringtoniae*, *R. goodenoughii* and *R. herzogii* and exhibited flowers with longer, narrower corolla tubes and asymmetry in reflexing of the upper vs lower petals. The second shift was shared by *R. beyerinckianum*, *R. calignis*, *R. dielsianum*, *R. leptanthum*, *R. phaeochitum* and *R. rarum* and confirmed the origin of a second novel form of bilateral symmetry in clade Euvireya D (clade 7), which exhibited curved corolla tubes and longer upper petal lobes than lower petal lobes (Fig. 7c). When we included these side views, we observed that all taxa sampled had some form of asymmetry between the upper and lower parts of the flower due to differential reflexing or length of lobes (Fig. 7c). Therefore, taking a side view of flowers showed that the ancestral phenotype of sect. *Schistanthe* may have been a bilaterally symmetric flower that also exhibited asymmetry between the upper and lower parts of the flower, followed by other types of asymmetry between the upper and lower parts of the flower arising within clades.

## Discussion

We used reduced genome‐wide sequencing from 114 members of *Rhododendron* sect. *Schistanthe* and revealed the evolution of floral diversity across the tropical radiation of *Rhododendron* in Southeast Asia by using morphometrics of corollas to characterise variation in corolla symmetry. Corolla symmetry in sect. *Schistanthe* is a spectrum of bilateral symmetry that exhibits variation in asymmetry between the upper and lower parts of the corolla with respect to petal curvature, number, angle, length and/or reflexing. We suggest that the ancestral corolla phenotype for sect. *Schistanthe* may have been bilaterally symmetrical when taking into account both front and side views of the corolla. The New Guinean radiation of sect. *Schistanthe* (Euvireya D, clade 7) evolved to occupy the largest corolla morphospace in the shortest amount of time and witnessed three shifts in floral evolution. This included the origin of two unique types of bilateral symmetry, in which corollas either show doubling of lateral petals and wider angles between upper vs lower petal lobes or corolla tubes become curved with longer upper than lower petal lobes.

### Phylogenetic history of *Rhododendron* sect. *Schistanthe* is confounded by recent speciation, hybridisation and introgression

Understanding the evolution of floral diversity in sect. *Schistanthe* is impeded by phylogenetic complexity and uncertainty. Prior phylogenetic studies of sect. *Schistanthe* based on cpDNA (Kurashige *et al*., 2001; Brown *et al*., 2006a) or several nuclear genes (*RPB2‐D*, *RPB2‐I*, *RPC1*) (Goetsch *et al*., 2011) showed sect. *Schistanthe* as polyphyletic, whereas studies based on ITS showed monophyly for the group (Brown *et al*., 2006b). In all our phylogenomic analyses, we recovered a monophyletic sect. *Schistanthe*. The presence of idioblasts, cells that are likely to function as a water buffering system (Tulyananda & Nilsen, 2017), in leaves has been identified as a morphological synapomorphy for sect. *Schistanthe* (Nilsen, 2003; Nilsen & Scheckler, 2003) and supports its monophyly. However, we did find conflict in the monophyly of subsect. *Pseudovireya* that could be due to introgression with a relative outside of sect. *Schistanthe* that we did not sample. In addition, we confirmed the monophyly of the other three subsections in the group: *Discovireya*, *Malayovireya* and *Euvireya*. We reconstructed subsect. *Pseudovireya* as sister to the other subsections, whereas the nuclear gene phylogeny of Goetsch *et al*. (2011) reconstructed subsect. *Discovireya* as sister to the other subsections. Based on our phylogenetic reconstructions and subsect. *Pseudovireya*'s extant geographic distribution in India and China (Fig. 5) (Argent, 2015), *Pseudovireya* is likely to be sister to the rest of sect. *Schistante* and the probable origin for sect. *Schistanthe*.

We found evidence of introgression both within and between clades of sect. *Schistanthe*, which may in part be a cause of phylogenetic conflict and uncertainty. Hybridisation has previously been reported between members within clades (Sleumer, 1973; Stevens, 1974; Argent, 1985, 2015; Cruttwell, 1988; Argent *et al*., 2007; Craven *et al*., 2011) and between clades (Sleumer, 1966, 1973; Argent, 1985, 2015; Cruttwell, 1988; Argent *et al*., 2007). Two potential introgression events we detected between clades that were likely to be the cause of topological conflict were (1) between *R. longiflorum* (clade Euvireya C/clade 6) and the majority of clade Euvireya D (clade 7), which potentially affected the monophyly of clade Euvireya C (clade 6), and (2) a weak signal of introgression between clades Euvireya B (clade 5) and Euvireya D (clade 7), potentially affecting the position and monophyly of clade Euvireya B (clade 5). We also detected many cases of introgression within clades. Species from sect. *Schistanthe* hybridise easily in cultivation as long as species have similar style lengths (Williams & Rouse, 1988; Rouse *et al*., 1993), with the exception of members from clade Pseudovireya (clade 1), which are incompatible in artificial crosses that have been attempted (Rouse, 1985; Rouse *et al*., 1993). All chromosome counts to date are uniformly 2*n* = 26 (Janaki Ammal *et al*., 1950; Jones & Brighton, 1972; Atkinson *et al*., 2000), suggesting that polyploidy is not a barrier between species and indicating homoploid hybridisation within the group. Therefore, hybridisation is widely reported in *Rhododendron*, and the recent rapid diversification of species may have been stimulated by hybridisation and introgression between species from across the group. Further genomic sequencing of sympatric taxa may shed light on the mode and tempo of hybridisation and whether adaptive introgression underlies the evolution of novel floral morphologies.

### Floral radiation in *Rhododendron* sect. *Schistanthe*

We found a spectrum of bilateral symmetry in sect. *Schistanthe*. This variation is based on asymmetry between the upper and lower parts of the corolla with respect to curvature of petals, number of petals, angles between petal lobes, length of lobes and/or reflexing of lobes. We inferred a likely ancestral phenotype of bilateral symmetry for sect. *Schistanthe*, and we estimated that all sampled taxa were bilaterally symmetrical when taking into account the side view of corollas. However, when only examining the front view of corollas, different types of radial and bilateral symmetry can be recognised. Therefore, different symmetry types have evolved in the group, and symmetry here is more of a continuum rather than two different states (radial or bilateral). Our results contrast with previous work that used a more subjective coding of symmetry and only the front view of corollas, which inferred a radially symmetric corolla for sect. *Schistanthe* (Berry *et al*., 2018). Future studies are needed to comprehensively evaluate the ancestral phenotype for sect. *Schistanthe* taking into account suitable sampling of outgroups, quantitative three‐dimensional views of the flower and species tree methods that account for introgression.

In general, the low proportion of variance explained by each PC in our morphometric analyses showed the complexity of corolla morphology in sect. *Schistanthe*. Other studies that have attempted to examine corolla shape in the group have also found it to be highly homoplasious (Brown *et al*., 2006a). Variation and overlap among clades Euvireya A (clade 4), Euvireya C (clade 6) and Euvireya D (clade 7) could have potentially resulted from introgression facilitating convergent evolution, as we found evidence for introgression among these clades. Nevertheless, we observed species from the New Guinean clade (Euvireya D, clade 7) expanding into new morphological space distinct from other clades. We recognised two novel forms of bilateral symmetry unique to this clade (Fig. S14), including 38 species in New Guinea that have an ornithophilous type of flower with a curved corolla tube and adaxially positioned stamens and style (Stevens, 1982, 1985) and 10 species from New Guinea that have white, sweet‐scented flowers thought to be bat‐pollinated with seven corolla lobes, 14 stamens and a seven‐lobed stigma (Sleumer, 1966; van Royen & Kores, 1982; Stevens, 1982, 1985; Rouse, 1985; Cruttwell, 1988; Argent, 2015). Both of these bilaterally symmetric flower types were considered by Stevens (1982) to be unique for Ericaceae. We found an expansion of clade Euvireya D (clade 7) into new corolla spaces that probably reflects morphological radiation in this New Guinean group. Cruttwell (1988) hypothesised that rhododendrons in New Guinea were in a ‘rapid state of evolution’ due to their great morphological variation, local occurrence of many species, high degree of endemism and potential for hybrid swarms. In our study, we found evidence for this great morphological variation, local occurrence of many species and extensive hybridisation in this recent radiation.

Pollination information for sect. *Schistanthe* is patchy, with visitor observations (Stevens, 1976; Argent, 1985; Cruttwell, 1988; Argent *et al*., 2007), but no studies confirming these animals as pollinators. Flowers from the section are presented in a variety of ways: erect to horizontal to pendent (Fig. 1) (Argent, 2015). Sunbirds (Nectariniidae), honeyeaters (Meliphagidae), butterflies, bees and other groups in the Old World appear to be pollinators (reviewed in Stevens, 1976; Stevens *et al*., 2004), while bird pollination seems to predominate in species at high altitudes (>3000 m) in New Guinea (Stevens, 1976). Wade & McVean (1969) considered the wide range in corolla tube lengths across sect. *Schistanthe* as an adaptation to diverse honeyeaters, with long bills visiting species with long, curved corolla tubes and adaxially positioned stamens, whereas shorter billed members visited species with shorter tubes (Wade & McVean, 1969). However, further west in the Malay Archipelago, sunbirds are the main group of nectar‐eating birds and have been observed visiting sect. *Schistanthe* in this region (Stevens, 1976). Species that are likely to be pollinated by butterflies, sphingids and bats are found more commonly below 3000 m (reviewed in Stevens *et al*., 2004). Butterflies have been observed visiting species with large, orange‐yellow, broadly funnel‐shaped flowers or red/yellow‐flowered species, such as *R*. *christi* and *R. javanicum* (Stevens, 1976; Argent, 1985; Cruttwell, 1988), but no observations have been recorded of visits by moths or bats. Despite the observations made, more experimental studies are needed to determine whether pollinator‐mediated selection has contributed to this wide morphological variation of corollas within sect. *Schistanthe*.

## Future directions

We used morphometrics of both the front and side views of corollas to characterise and understand symmetry evolution in sect. *Schistanthe*. We used side views of corolla outlines for the first time to examine corolla symmetry variation in angiosperms and, in so doing, we obtained a more holistic view of symmetry. Extending this method to examine floral symmetry evolution in other angiosperms will allow future studies to quantify variation more accurately. In addition, using morphometrics to characterise changes in floral symmetry may reveal genomic differences underlying different transitions in symmetry.

Different genetic components could be controlling each component of floral symmetry observed in sect. *Schistanthe*. For example, the transcription factor *CYCLOIDEA* is likely to be involved in the asymmetrical growth of petals and petal number (Luo *et al*., 1996, 1999; Preston *et al*., 2014; Berger *et al*., 2017; Hsu *et al*., 2017; Ramage *et al*., 2021). Therefore, this group is an ideal system to study the genetic underpinnings involved in variation of corolla symmetry and its associated transitions. Other *Rhododendron* species outside of sect. *Schistanthe* also deviate from the normal five‐merous flowers of the genus but appear radially symmetrical with fewer than (e.g. former *Menziesia*) or greater than five petals. Therefore, morphometric analyses of the corolla should be expanded to the entire genus in future studies for a more holistic view of the flower. Three‐dimensional imaging using methods such as microcomputed tomography (Wang *et al*., 2015) will be useful for capturing overall floral symmetry variation more accurately.

## Author contributions

VLS, RK, BDH and ADT designed the research. VLS, RK, ER and ADT performed the research by conducting data collection, analyses and/or interpretation. VLS, RK, ER and ADT wrote the manuscript.

## Supporting information


**Fig. S1** Clustering threshold series for *Rhododendron* sect. *Schistanthe* RAD‐seq data.
**Fig. S2** Ipyrad parameter settings for *Rhododendron* sect. *Schistanthe* RAD‐seq data.
**Fig. S3** RAxML topology of *Rhododendron* sect. *Schistante* and outgroups that was generated from the min4 dataset, which required four samples per locus.
**Fig. S4** RAxML topology of *Rhododendron* sect. *Schistante* and outgroups that was generated from the min37 dataset, which required 37 samples per locus.
**Fig. S5** RAxML topology of *Rhododendron* sect. *Schistante* and outgroups that was generated from the min74 dataset, which required 74 samples per locus.
**Fig. S6** RAxML topology of *Rhododendron* sect. *Schistante* and outgroups that was generated from the min111 dataset, which required 111 samples per locus.
**Fig. S7** SVDQuartets consensus topology of *Rhododendron* sect. *Schistante* that was generated from the min37 dataset, which required 37 samples per locus.
**Fig. S8** SVDQuartets consensus topology of *Rhododendron* sect. *Schistante* that was generated from the min74 dataset, which required 74 samples per locus.
**Fig. S9** SVDQuartets consensus topology of *Rhododendron* sect. *Schistante* that was generated from the min111 dataset, which required 111 samples per locus.
**Fig. S10** Heatmap of *D*_min_‐statistics in *Rhododendron* sect. *Schistanthe*.
**Fig. S11** Heatmap of *D*_tree_‐statistics in *Rhododendron* sect. *Schistanthe*.
**Fig. S12** Panel of outlines used to reconstruct floral morphospaces for *Rhododendron* sect. *Schistanthe*.
**Fig. S13** Morphospace variation and clustering of corollas from *Rhododendron* sect. *Schistanthe* using principal component analysis (PCA).
**Fig. S14** Morphospace variation of corollas from *Rhododendron* sect. *Schistanthe* using principal component analysis (PCA) and flower colour.
**Methods S1** Library preparation.
**Methods S2** Data processing.
**Methods S3** Phylogenetic analyses.
**Methods S4** Introgression analyses.
**Methods S5** Molecular dating.
**Methods S6** Morphometric analyses.
**Table S1** Samples and vouchers used in this study.
**Table S2** Characteristics for each dataset used in *Rhododendron* sect. *Schistanthe* analyses.
Please note: Wiley Blackwell are not responsible for the content or functionality of any Supporting Information supplied by the authors. Any queries (other than missing material) should be directed to the *New Phytologist* Central Office.

## Data Availability

All data that support the findings of this study are openly available in the following repositories. Raw sequencing data are deposited in the National Center for Biotechnology Information Sequence Read Archive under BioProjects PRJNA758441 (https://www.ncbi.nlm.nih.gov/sra/PRJNA758441) and PRJNA769944 (https://www.ncbi.nlm.nih.gov/sra/PRJNA769944). The sequence alignments, tree files and morphological data and scripts for morphometric analyses are available in Dryad (10.5061/dryad.47d7wm3f4). Scripts used for processing phylogenetic data are available at https://github.com/vsoza/vireya‐RAD, doi: 10.5281/zenodo.5760113.
