# Supplementary material for: The symmetry spectrum in a hybridising, tropical group of rhododendrons
Source: New Phytol. 2022 Mar 29;234(4):1491–506. doi: 10.1111/nph.18083 (PMC9313591; doi:10.1111/nph.18083)
Supplement: Supplementary file 1 — Fig. S1 Clustering threshold series for Rhododendron sect. Schistanthe RAD‐seq data. Fig. S2 Ipyrad parameter settings for Rhododendron sect. Schistanthe RAD‐seq data. Fig. S3 RAxML topology of Rhododendron sect. Schistante and outgroups that was generated from the min4 dataset, which required four samples per locus. Fig. S4 RAxML topology of Rhododendron sect. Schistante and outgroups that was generated from the min37 dataset, which required 37 samples per locus. Fig. S5 RAxML topology of Rhododendron sect. Schistante and outgroups that was generated from the min74 dataset, which required 74 samples per locus. Fig. S6 RAxML topology of Rhododendron sect. Schistante and outgroups that was generated from the min111 dataset, which required 111 samples per locus. Fig. S7 SVDQuartets consensus topology of Rhododendron sect. Schistante that was generated from the min37 dataset, which required 37 samples per locus. Fig. S8 SVDQuartets consensus topology of Rhododendron sect. Schistante that was generated from the min74 dataset, which required 74 samples per locus. Fig. S9 SVDQuartets consensus topology of Rhododendron sect. Schistante that was generated from the min111 dataset, which required 111 samples per locus. Fig. S10 Heatmap of D min‐statistics in Rhododendron sect. Schistanthe. Fig. S11 Heatmap of D tree‐statistics in Rhododendron sect. Schistanthe. Fig. S12 Panel of outlines used to reconstruct floral morphospaces for Rhododendron sect. Schistanthe. Fig. S13 Morphospace variation and clustering of corollas from Rhododendron sect. Schistanthe using principal component analysis (PCA). Fig. S14 Morphospace variation of corollas from Rhododendron sect. Schistanthe using principal component analysis (PCA) and flower colour. Methods S1 Library preparation. Methods S2 Data processing. Methods S3 Phylogenetic analyses. Methods S4 Introgression analyses. Methods S5 Molecular dating. Methods S6 Morphometric analyses. Table S1 Samples and vouchers used in this study. Table [file NPH-234-1491-s001.pdf]

New Phytologist **Supporting Information**

Article title: The symmetry spectrum in a hybridizing, tropical group of rhododendrons
Authors: Valerie L. Soza, Ricardo Kriebel, Elizabeth Ramage, Benjamin D. Hall, Alex D. Twyford
Article acceptance date: 23 January 2022

The following Supporting Information is available for this article:

Fig. S1 Clustering threshold series for *Rhododendron* sect. *Schistanthe* RAD-seq data.
Y-axis shows mean (+/- SD) percentage of clusters per sample that were assembled according to Ilut *et al.* (2014) method. X-axis shows maximum pairwise distances allowed between reads within a cluster. Blue data represent single haplotype clusters (homozygous loci), red data represent three or more haplotype clusters (paralogous loci), and green data represent two-haplotype clusters (heterozygous loci). A maximum distance of nine was determined as the optimum clustering threshold to maximize two-haplotype clusters and minimize single haplotype clusters.

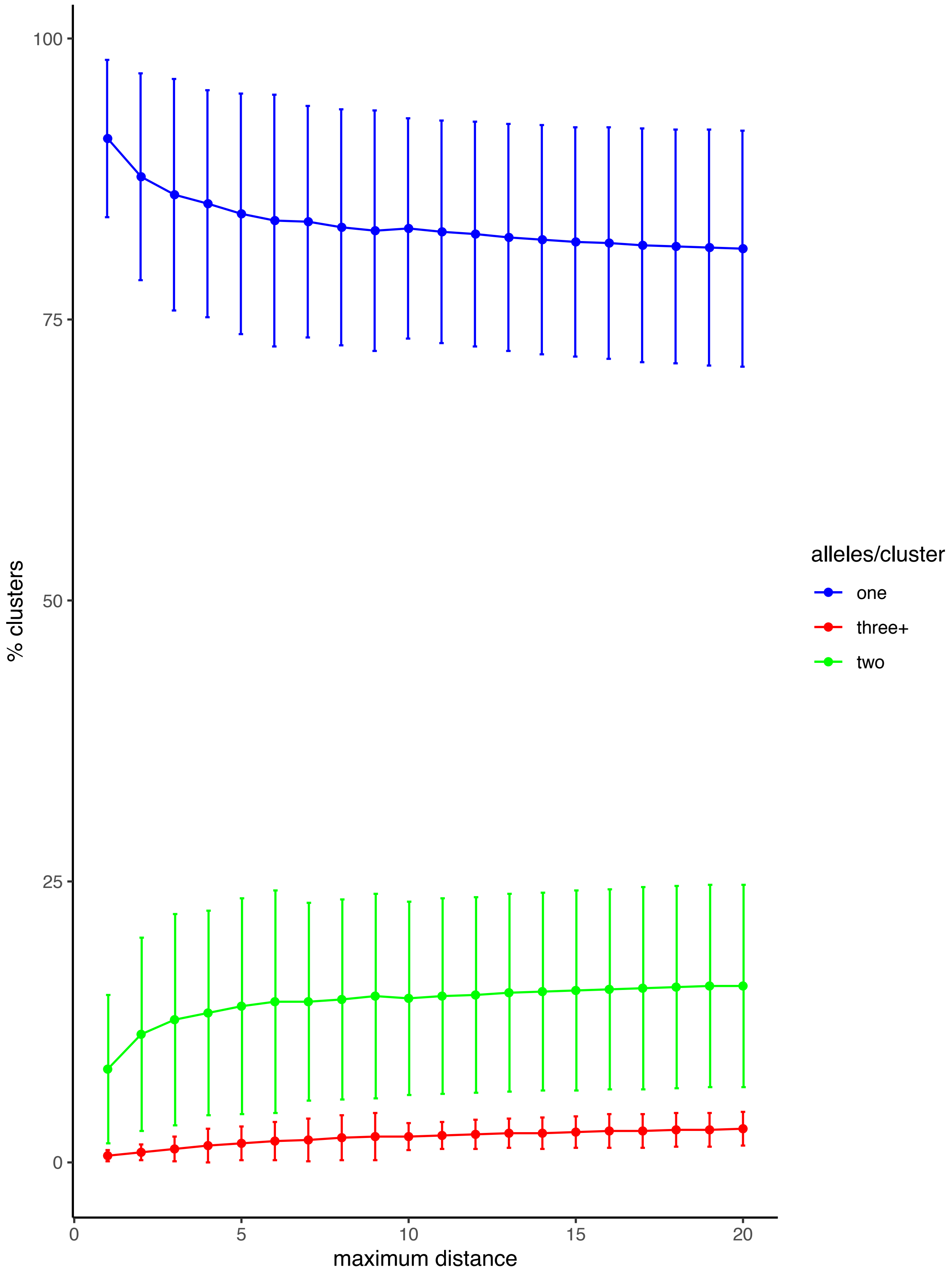

100
75
50
25
0
% clusters
0
5
10
15
20
maximum distance
alleles/cluster
one
three+
two

Fig. S2 *ipyrad* parameter settings for *Rhododendron* sect. *Schistanthe* RAD-seq data.
A range of settings for a variety of *ipyrad* parameters were experimented with to identify the optimal setting based on number of retained loci. (a) maximum indels per locus. (b) maximum SNPs per locus. (c) maximum shared heterozygosity per locus. (d) minimum sample coverage per locus.

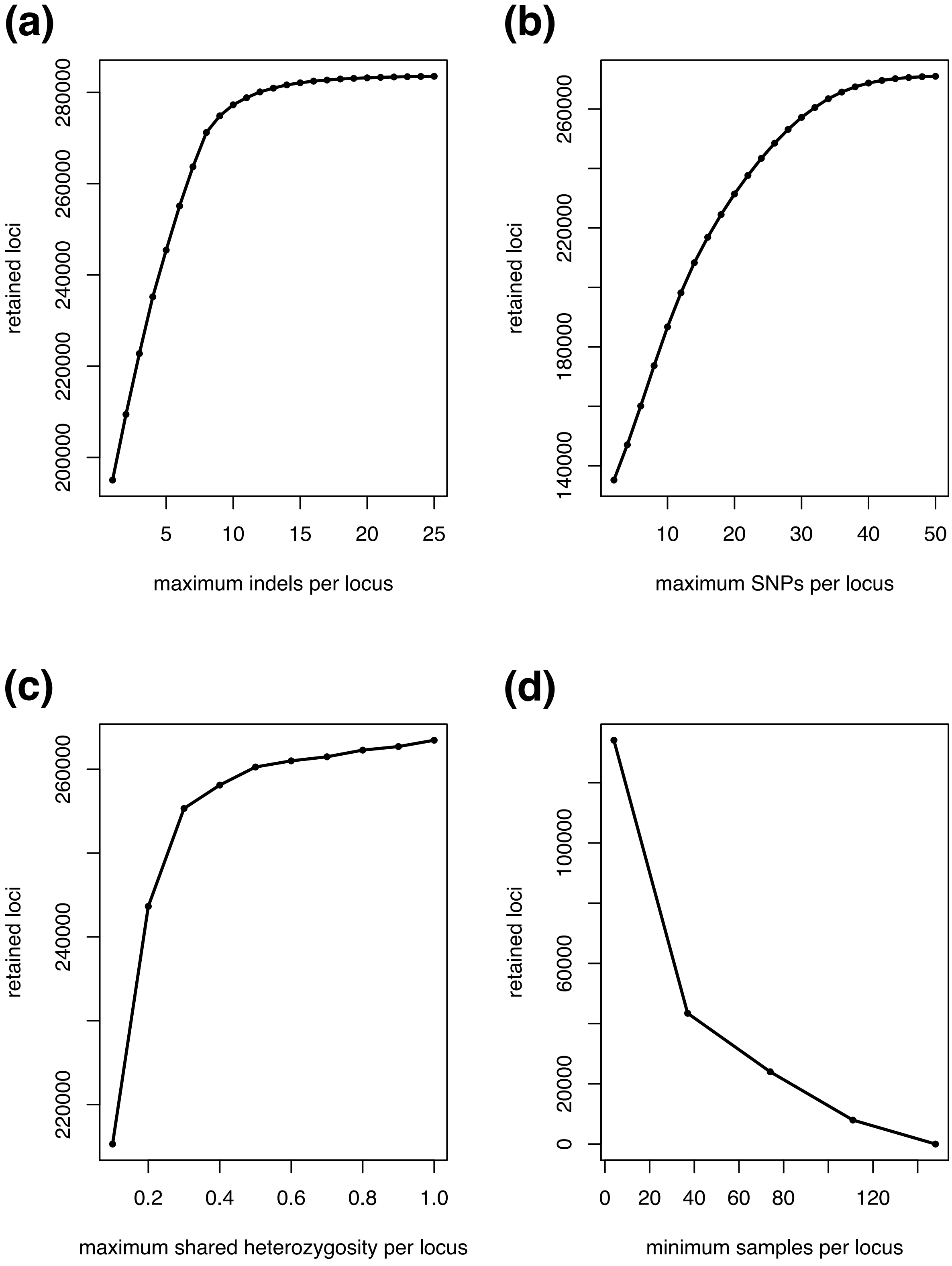
(a)
retained loci
200000
220000
240000
260000
280000
5
10
15
20
25
maximum indels per locus
(b)
retained loci
140000
180000
220000
260000
10
20
30
40
50
maximum SNPs per locus
(c)
retained loci
220000
240000
260000
0.2
0.4
0.6
0.8
1.0
maximum shared heterozygosity per locus
(d)
retained loci
0
20000
60000
100000
0
20
40
60
80
120
minimum samples per locus

Fig. S3 RAxML topology of *Rhododendron* sect. *Schistante* and outgroups that was generated from the min4 dataset, which required four samples per locus.

Support values at nodes for *Rhododendron* are shown in the following order: Quartet Concordance/Quartet Differential/Quartet Informativeness/bootstrap. Seven main clades (1-7) were identified in the study and referred to based on subsectional classification: Pseudovireya, Discovireya, Malayovireya, Euvireya A, Euvireya B, Euvireya C, and Euvireya D, respectively.

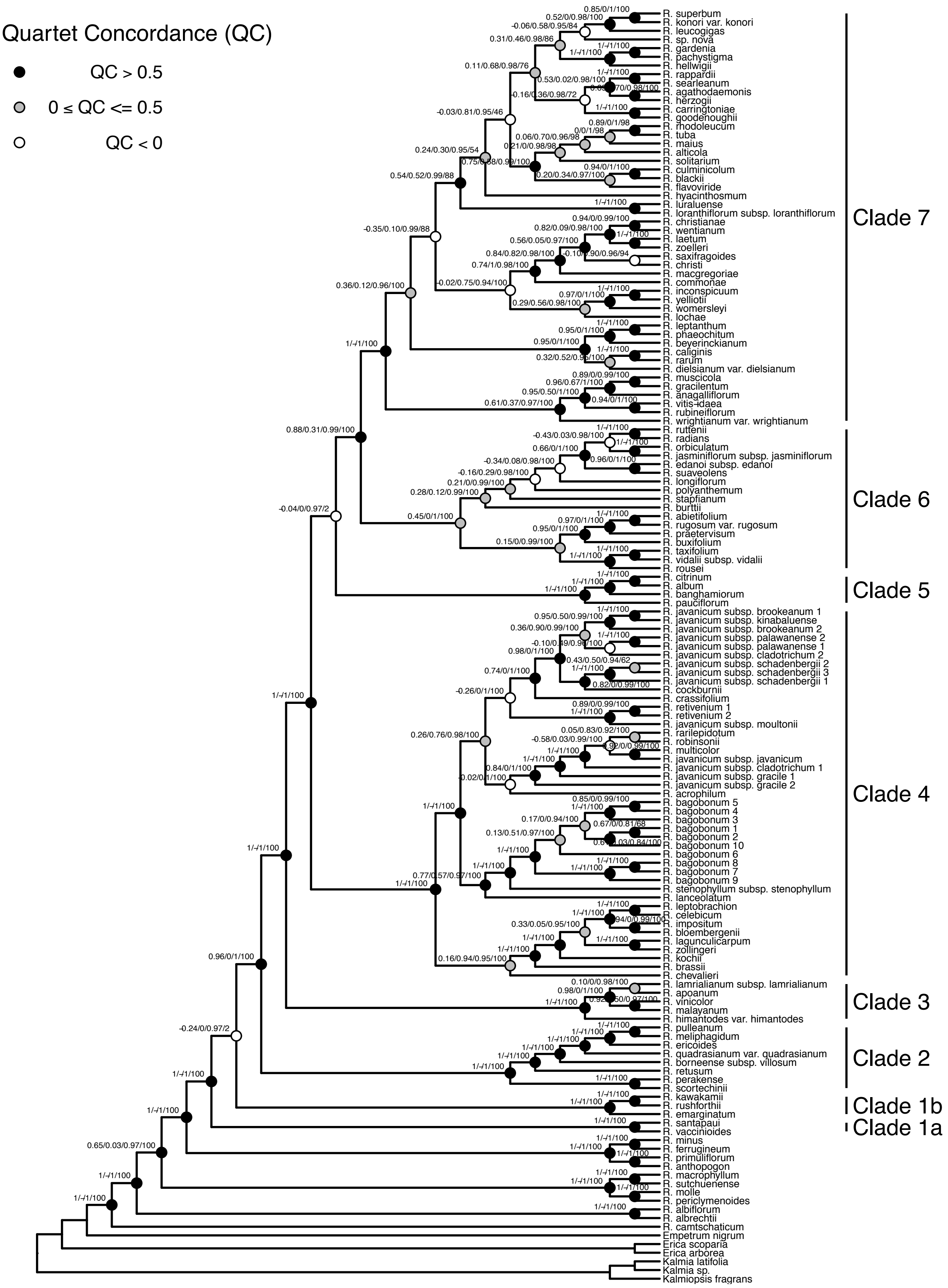

Quartet Concordance (QC)
QC > 0.5
0 ≤ QC <= 0.5
QC < 0
R. superbum
R. konori var. konori
R. leucogigas
R. sp. nova
R. gardenia
R. pachystigma
R. hellwigii
R. rappardii
R. searleanum
R. agathodaemonis
R. herzogii
R. carringtoniae
R. goodenoughii
R. rhodoleucum
R. tuba
R. maius
R. alticola
R. solitarium
R. culminicolum
R. blackii
R. flavoviride
R. hyacinthosmum
R. luraluense
R. loranthiflorum subsp. loranthiflorum
R. christianae
R. wentianum
R. laetum
R. zoelleri
R. saxifragoides
R. christi
R. macgregoriae
R. commonae
R. inconspicuum
R. yelliotii
R. womersleyi
R. lochae
R. leptanthum
R. phaeochitum
R. beyerinckianum
R. caliginis
R. rarum
R. dielsianum var. dielsianum
R. muscicola
R. gracilentum
R. anagalliflorum
R. vitis-idaea
R. rubineiflorum
R. wrightianum var. wrightianum
R. ruttenii
R. radians
R. orbiculatum
R. jasminiflorum subsp. jasminiflorum
R. edanoi subsp. edanoi
R. suaveolens
R. longiflorum
R. polyanthemum
R. stapfianum
R. burttii
R. abietifolium
R. rugosum var. rugosum
R. praetervisum
R. buxifolium
R. taxifolium
R. vidalii subsp. vidalii
R. rousei
R. citrinum
R. album
R. banghamiorum
R. pauciflorum
R. javanicum subsp. brookeanum 1
R. javanicum subsp. kinabaluense
R. javanicum subsp. brookeanum 2
R. javanicum subsp. palawanense 2
R. javanicum subsp. palawanense 1
R. javanicum subsp. cladotrichum 2
R. javanicum subsp. schadenbergii 2
R. javanicum subsp. schadenbergii 3
R. javanicum subsp. schadenbergii 1
R. cockburnii
R. crassifolium
R. retivenium 1
R. retivenium 2
R. javanicum subsp. moultonii
R. rarilepidotum
R. robinsonii
R. multicolor
R. javanicum subsp. javanicum
R. javanicum subsp. cladotrichum 1
R. javanicum subsp. gracile 1
R. javanicum subsp. gracile 2
R. acrophilum
R. bagobonum 5
R. bagobonum 4
R. bagobonum 3
R. bagobonum 1
R. bagobonum 2
R. bagobonum 10
R. bagobonum 6
R. bagobonum 8
R. bagobonum 7
R. bagobonum 9
R. stenophyllum subsp. stenophyllum
R. lanceolatum
R. leptobrachion
R. celebicum
R. impositum
R. bloembergenii
R. lagunculicarpum
R. zollingeri
R. kochii
R. brassii
R. chevalieri
R. lamrialianum subsp. lamrialianum
R. apoanum
R. vinicolor
R. malayanum
R. himantodes var. himantodes
R. pulleanum
R. meliphagidum
R. ericoides
R. quadrasianum var. quadrasianum
R. borneense subsp. villosum
R. retusum
R. perakense
R. scortechinii
R. kawakamii
R. rushforthii
R. emarginatum
R. santapaui
R. vaccinioides
R. minus
R. ferrugineum
R. primuliflorum
R. anthopogon
R. macrophyllum
R. sutchuenense
R. molle
R. periclymenoides
R. albiflorum
R. albrechtii
R. camtschaticum
Empetrum nigrum
Erica scoparia
Erica arborea
Kalmia latifolia
Kalmia sp.
Kalmiopsis fragrans
Clade 7
Clade 6
Clade 5
Clade 4
Clade 3
Clade 2
Clade 1b
Clade 1a

Fig. S4 RAxML topology of *Rhododendron* sect. *Schistante* and outgroups that was generated from the min37 dataset, which required 37 samples per locus.

Support values at nodes for *Rhododendron* are shown in the following order: Quartet Concordance/Quartet Differential/Quartet Informativeness/bootstrap. Seven main clades (1-7) were identified in the study and referred to based on subsectional classification: Pseudovireya, Discovireya, Malayovireya, Euvireya A, Euvireya B, Euvireya C, and Euvireya D, respectively.

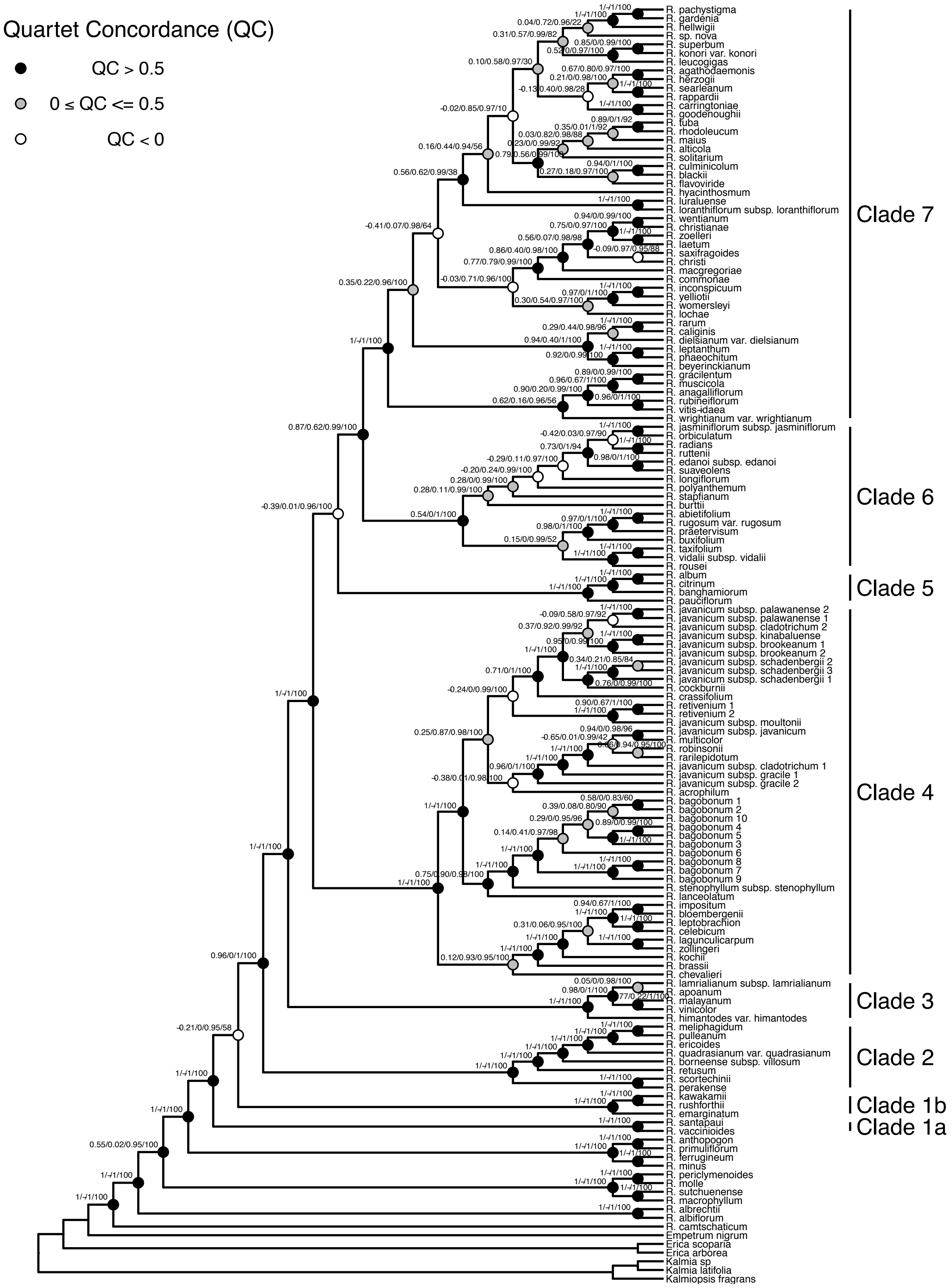

Quartet Concordance (QC)
QC > 0.5
0 ≤ QC <= 0.5
QC < 0
Clade 7
Clade 6
Clade 5
Clade 4
Clade 3
Clade 2
Clade 1b
Clade 1a
R. pachystigma
R. gardenia
R. hellwigii
R. sp. nova
R. superbum
R. konori var. konori
R. leucogigas
R. agathodaemonis
R. herzogii
R. searleanum
R. rappardii
R. carringtoniae
R. goodenoughii
R. tuba
R. rhodoleucum
R. maius
R. alticola
R. solitarium
R. culminicolum
R. blackii
R. flavoviride
R. hyacinthosmum
R. luraluense
R. loranthiflorum subsp. loranthiflorum
R. wentianum
R. christianae
R. zoelleri
R. laetum
R. saxifragoides
R. christi
R. macgregoriae
R. commonae
R. inconspicuum
R. yelliotii
R. womersleyi
R. lochae
R. rarum
R. caliginis
R. dielsianum var. dielsianum
R. leptanthum
R. phaeochitum
R. beyerinckianum
R. gracilentum
R. muscicola
R. anagalliflorum
R. rubineiflorum
R. vitis-idaea
R. wrightianum var. wrightianum
R. jasminiflorum subsp. jasminiflorum
R. orbiculatum
R. radians
R. ruttenii
R. edanoi subsp. edanoi
R. suaveolens
R. longiflorum
R. polyanthemum
R. stapfianum
R. burttii
R. abietifolium
R. rugosum var. rugosum
R. praetervisum
R. buxifolium
R. taxifolium
R. vidalii subsp. vidalii
R. rousei
R. album
R. citrinum
R. banghamiorum
R. pauciflorum
R. javanicum subsp. palawanense 2
R. javanicum subsp. palawanense 1
R. javanicum subsp. cladotrichum 2
R. javanicum subsp. kinabaluense
R. javanicum subsp. brookeanum 1
R. javanicum subsp. brookeanum 2
R. javanicum subsp. schadenbergii 2
R. javanicum subsp. schadenbergii 3
R. javanicum subsp. schadenbergii 1
R. cockburnii
R. crassifolium
R. retivenium 1
R. retivenium 2
R. javanicum subsp. moultonii
R. javanicum subsp. javanicum
R. multicolor
R. robinsonii
R. rarilepidotum
R. javanicum subsp. cladotrichum 1
R. javanicum subsp. gracile 1
R. javanicum subsp. gracile 2
R. acrophilum
R. bagobonum 1
R. bagobonum 2
R. bagobonum 10
R. bagobonum 4
R. bagobonum 5
R. bagobonum 3
R. bagobonum 6
R. bagobonum 8
R. bagobonum 7
R. bagobonum 9
R. stenophyllum subsp. stenophyllum
R. lanceolatum
R. impositum
R. bloembergenii
R. leptobrachion
R. celebicum
R. lagunculicarpum
R. zollingeri
R. kochii
R. brassii
R. chevalieri
R. lamrialianum subsp. lamrialianum
R. apoanum
R. malayanum
R. vinicolor
R. himantodes var. himantodes
R. meliphagidum
R. pulleanum
R. ericoides
R. quadrasianum var. quadrasianum
R. borneense subsp. villosum
R. retusum
R. scortechinii
R. perakense
R. kawakamii
R. rushforthii
R. emarginatum
R. santapaui
R. vaccinioides
R. anthopogon
R. primuliflorum
R. ferrugineum
R. minus
R. periclymenoides
R. molle
R. sutchuenense
R. macrophyllum
R. albrechtii
R. albiflorum
R. camtschaticum
Empetrum nigrum
Erica scoparia
Erica arborea
Kalmia sp
Kalmia latifolia
Kalmiopsis fragrans

Fig. S5 RAxML topology of *Rhododendron* sect. *Schistante* and outgroups that was generated from the min74 dataset, which required 74 samples per locus.
Support values at nodes for *Rhododendron* are shown in the following order: Quartet Concordance/Quartet Differential/Quartet Informativeness/bootstrap. Seven main clades (1-7) were identified in the study and referred to based on subsectional classification: Pseudovireya, Discovireya, Malayovireya, Euvireya A, Euvireya B, Euvireya C, and Euvireya D, respectively.

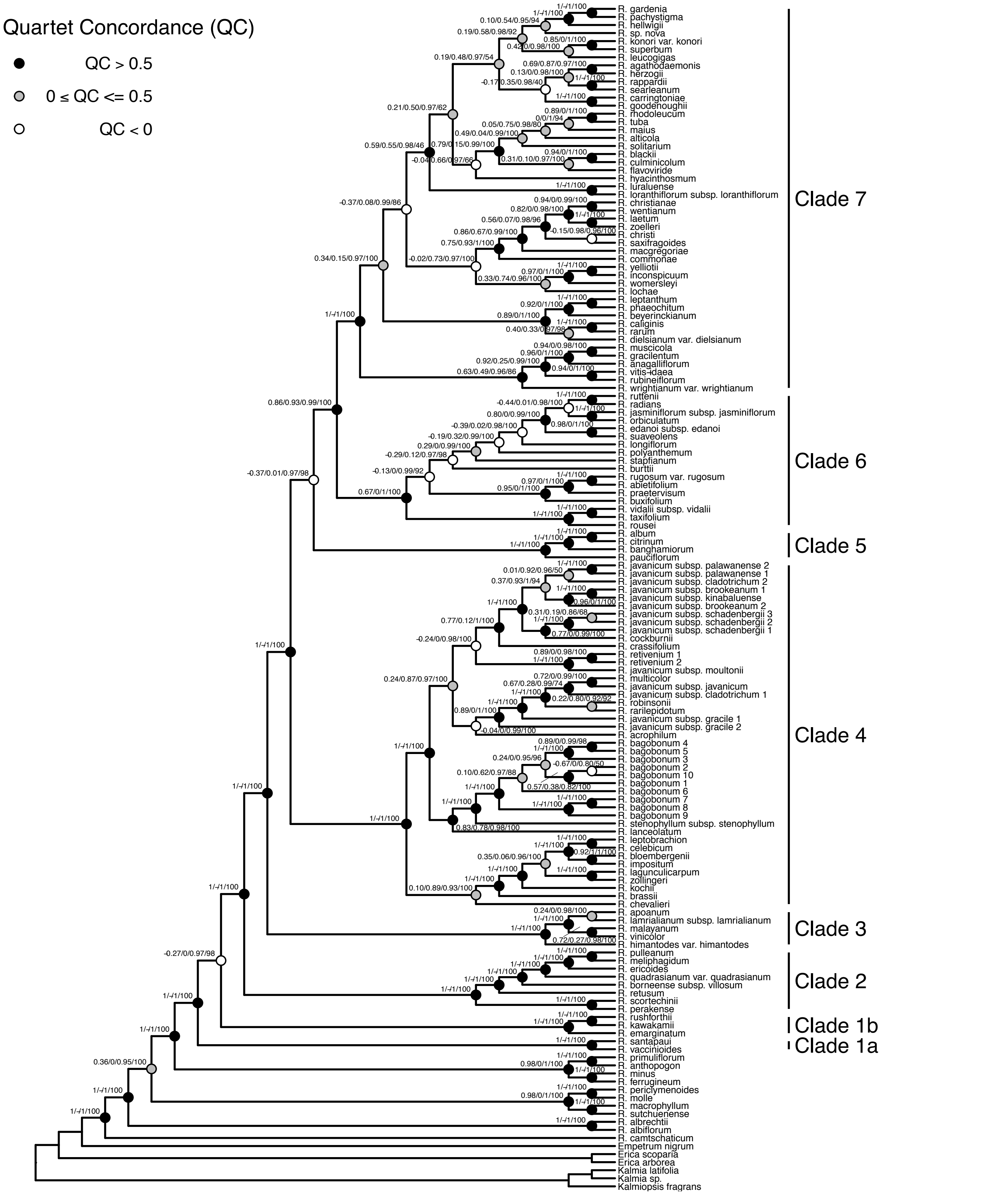
Quartet Concordance (QC)
QC > 0.5
0 ≤ QC <= 0.5
QC < 0
R. gardenia
R. pachystigma
R. hellwigii
R. sp. nova
R. konori var. konori
R. superbum
R. leucogigas
R. agathodaemonis
R. herzogii
R. rappardii
R. searleanum
R. carringtoniae
R. goodenoughii
R. rhodoleucum
R. tuba
R. maius
R. alticola
R. solitarium
R. blackii
R. culminicolum
R. flavoviride
R. hyacinthosmum
R. luraluense
R. loranthiflorum subsp. loranthiflorum
R. christianae
R. wentianum
R. laetum
R. zoelleri
R. christi
R. saxifragoides
R. macgregoriae
R. commonae
R. yelliotii
R. inconspicuum
R. womersleyi
R. lochae
R. leptanthum
R. phaeochitum
R. beyerinckianum
R. caliginis
R. rarum
R. dielsianum var. dielsianum
R. muscicola
R. gracilentum
R. anagalliflorum
R. vitis-idaea
R. rubineiflorum
R. wrightianum var. wrightianum
R. ruttenii
R. radians
R. jasminiflorum subsp. jasminiflorum
R. orbiculatum
R. edanoi subsp. edanoi
R. suaveolens
R. longiflorum
R. polyanthemum
R. stapfianum
R. burttii
R. rugosum var. rugosum
R. abietifolium
R. praetervisum
R. buxifolium
R. vidalii subsp. vidalii
R. taxifolium
R. rousei
R. album
R. citrinum
R. banghamiorum
R. pauciflorum
R. javanicum subsp. palawanense 2
R. javanicum subsp. palawanense 1
R. javanicum subsp. cladotrichum 2
R. javanicum subsp. brookeanum 1
R. javanicum subsp. kinabaluense
R. javanicum subsp. brookeanum 2
R. javanicum subsp. schadenbergii 3
R. javanicum subsp. schadenbergii 2
R. javanicum subsp. schadenbergii 1
R. cockburnii
R. crassifolium
R. retivenium 1
R. retivenium 2
R. javanicum subsp. moultonii
R. multicolor
R. javanicum subsp. javanicum
R. javanicum subsp. cladotrichum 1
R. robinsonii
R. rarilepidotum
R. javanicum subsp. gracile 1
R. javanicum subsp. gracile 2
R. acrophilum
R. bagobonum 4
R. bagobonum 5
R. bagobonum 3
R. bagobonum 2
R. bagobonum 10
R. bagobonum 1
R. bagobonum 6
R. bagobonum 7
R. bagobonum 8
R. bagobonum 9
R. stenophyllum subsp. stenophyllum
R. lanceolatum
R. leptobrachion
R. celebicum
R. bloembergenii
R. impositum
R. lagunculicarpum
R. zollingeri
R. kochii
R. brassii
R. chevalieri
R. apoanum
R. lamrialianum subsp. lamrialianum
R. malayanum
R. vinicolor
R. himantodes var. himantodes
R. pulleanum
R. meliphagidum
R. ericoides
R. quadrasianum var. quadrasianum
R. borneense subsp. villosum
R. retusum
R. scortechinii
R. perakense
R. rushforthii
R. kawakamii
R. emarginatum
R. santapaui
R. vaccinioides
R. primuliflorum
R. anthopogon
R. minus
R. ferrugineum
R. periclymenoides
R. molle
R. macrophyllum
R. sutchuenense
R. albrechtii
R. albiflorum
R. camtschaticum
Empetrum nigrum
Erica scoparia
Erica arborea
Kalmia latifolia
Kalmia sp.
Kalmiopsis fragrans
Clade 7
Clade 6
Clade 5
Clade 4
Clade 3
Clade 2
Clade 1b
Clade 1a

Fig. S6 RAxML topology of *Rhododendron* sect. *Schistante* and outgroups that was generated from the min111 dataset, which required 111 samples per locus.
Support values at nodes for *Rhododendron* are shown in the following order: Quartet Concordance/Quartet Differential/Quartet Informativeness/bootstrap. Seven main clades (1-7) were identified in the study and referred to based on subsectional classification: Pseudovireya, Discovireya, Malayovireya, Euvireya A, Euvireya B, Euvireya C, and Euvireya D, respectively.

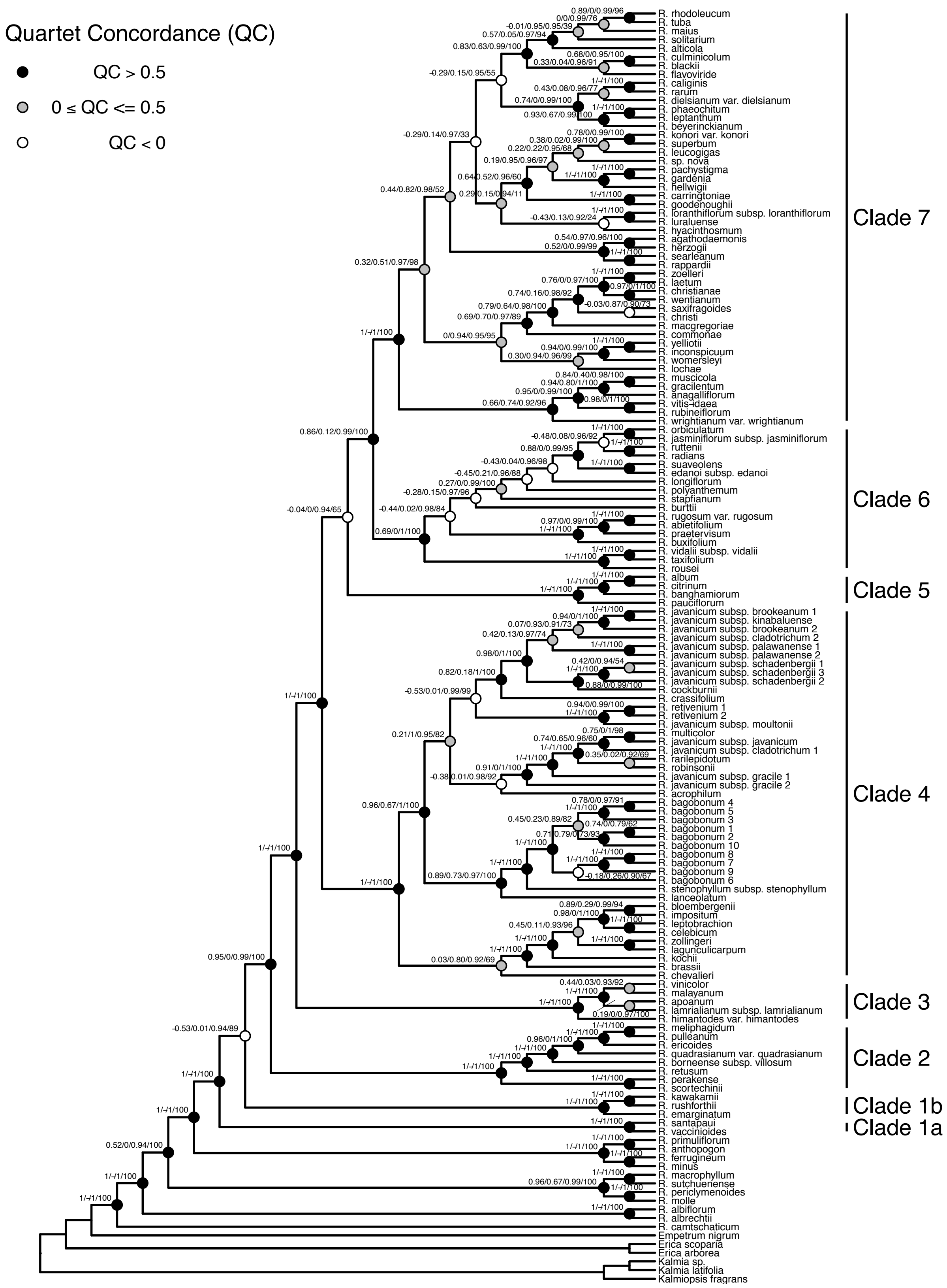

Quartet Concordance (QC)
QC > 0.5
0 ≤ QC <= 0.5
QC < 0
R. rhodoleucum
R. tuba
R. maius
R. solitarium
R. alticola
R. culminicolum
R. blackii
R. flavoviride
R. caliginis
R. rarum
R. dielsianum var. dielsianum
R. phaeochitum
R. leptanthum
R. beyerinckianum
R. konori var. konori
R. superbum
R. leucogigas
R. sp. nova
R. pachystigma
R. gardenia
R. hellwigii
R. carringtoniae
R. goodenoughii
R. loranthiflorum subsp. loranthiflorum
R. luraluense
R. hyacinthosmum
R. agathodaemonis
R. herzogii
R. searleanum
R. rappardii
R. zoelleri
R. laetum
R. christianae
R. wentianum
R. saxifragoides
R. christi
R. macgregoriae
R. commonae
R. yelliotii
R. inconspicuum
R. womersleyi
R. lochae
R. muscicola
R. gracilentum
R. anagalliflorum
R. vitis-idaea
R. rubineiflorum
R. wrightianum var. wrightianum
R. orbiculatum
R. jasminiflorum subsp. jasminiflorum
R. ruttenii
R. radians
R. suaveolens
R. edanoi subsp. edanoi
R. longiflorum
R. polyanthemum
R. stapfianum
R. burttii
R. rugosum var. rugosum
R. abietifolium
R. praetervisum
R. buxifolium
R. vidalii subsp. vidalii
R. taxifolium
R. rousei
R. album
R. citrinum
R. banghamiorum
R. pauciflorum
R. javanicum subsp. brookeanum 1
R. javanicum subsp. kinabaluense
R. javanicum subsp. brookeanum 2
R. javanicum subsp. cladotrichum 2
R. javanicum subsp. palawanense 1
R. javanicum subsp. palawanense 2
R. javanicum subsp. schadenbergii 1
R. javanicum subsp. schadenbergii 3
R. javanicum subsp. schadenbergii 2
R. cockburnii
R. crassifolium
R. retivenium 1
R. retivenium 2
R. javanicum subsp. moultonii
R. multicolor
R. javanicum subsp. javanicum
R. javanicum subsp. cladotrichum 1
R. rarilepidotum
R. robinsonii
R. javanicum subsp. gracile 1
R. javanicum subsp. gracile 2
R. acrophilum
R. bagobonum 4
R. bagobonum 5
R. bagobonum 3
R. bagobonum 1
R. bagobonum 2
R. bagobonum 10
R. bagobonum 8
R. bagobonum 7
R. bagobonum 9
R. bagobonum 6
R. stenophyllum subsp. stenophyllum
R. lanceolatum
R. bloembergenii
R. impositum
R. leptobrachion
R. celebicum
R. zollingeri
R. lagunculicarpum
R. kochii
R. brassii
R. chevalieri
R. vinicolor
R. malayanum
R. apoanum
R. lamrialianum subsp. lamrialianum
R. himantodes var. himantodes
R. meliphagidum
R. pulleanum
R. ericoides
R. quadrasianum var. quadrasianum
R. borneense subsp. villosum
R. retusum
R. perakense
R. scortechinii
R. kawakamii
R. rushforthii
R. emarginatum
R. santapaui
R. vaccinioides
R. primuliflorum
R. anthopogon
R. ferrugineum
R. minus
R. macrophyllum
R. sutchuenense
R. periclymenoides
R. molle
R. albiflorum
R. albrechtii
R. camtschaticum
Empetrum nigrum
Erica scoparia
Erica arborea
Kalmia sp.
Kalmia latifolia
Kalmiopsis fragrans
Clade 7
Clade 6
Clade 5
Clade 4
Clade 3
Clade 2
Clade 1b
Clade 1a

Fig. S7 SVDQuartets consensus topology of *Rhododendron* sect. *Schistante* that was generated from the min37 dataset, which required 37 samples per locus.
Support values at nodes for the ingroup are shown in the following order: Quartet Concordance/Quartet Differential/Quartet Informativeness/bootstrap. Seven main clades (1-7) were identified in the study and referred to based on subsectional classification: Pseudovireya, Discovireya, Malayovireya, Euvireya A, Euvireya B, Euvireya C, and Euvireya D, respectively.

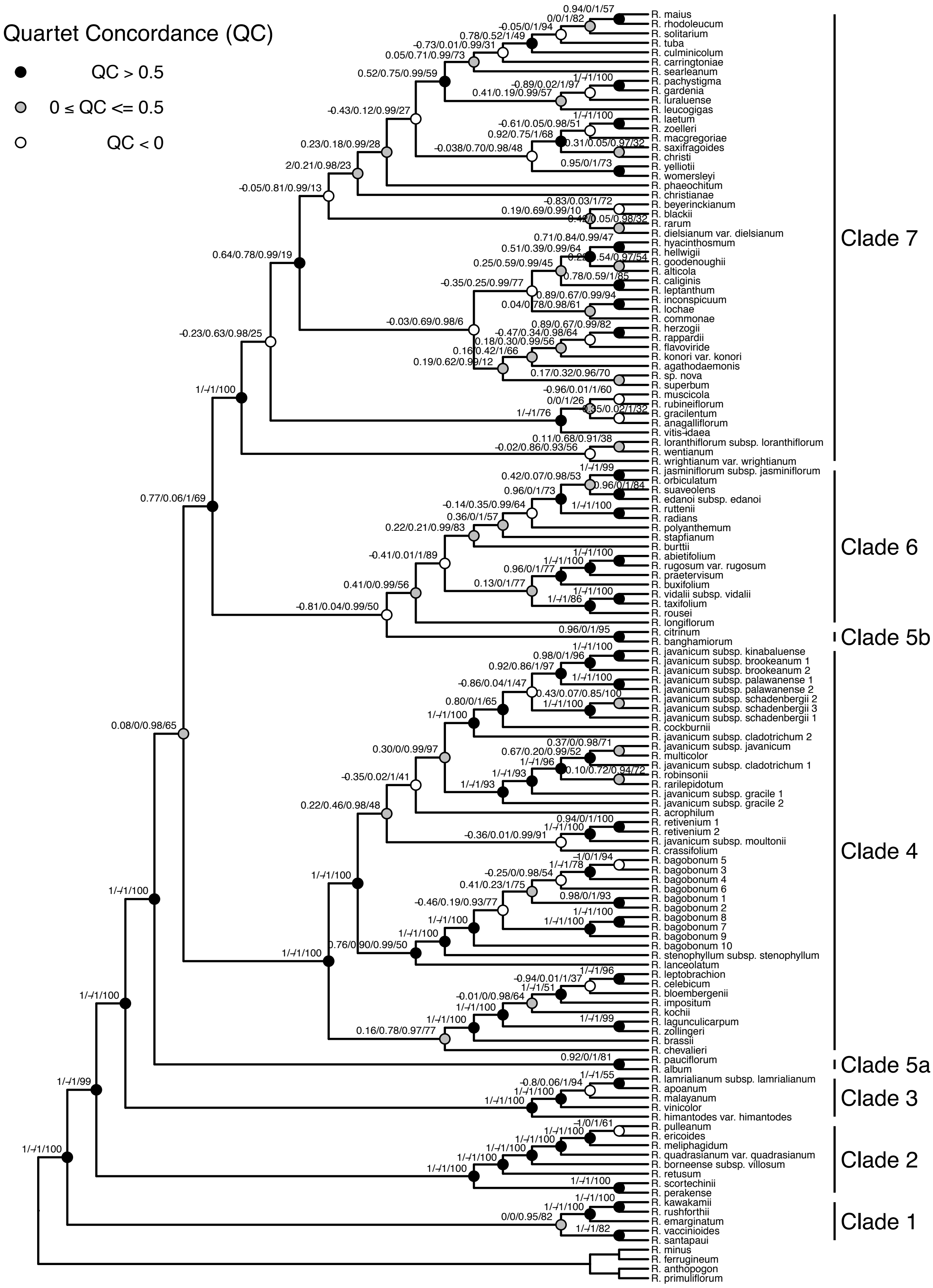
Quartet Concordance (QC)
QC > 0.5
0 ≤ QC <= 0.5
QC < 0
R. maius
R. rhodoleucum
R. solitarium
R. tuba
R. culminicolum
R. carringtoniae
R. searleanum
R. pachystigma
R. gardenia
R. luraluense
R. leucogigas
R. laetum
R. zoelleri
R. macgregoriae
R. saxifragoides
R. christi
R. yelliotii
R. womersleyi
R. phaeochitum
R. christianae
R. beyerinckianum
R. blackii
R. rarum
R. dielsianum var. dielsianum
R. hyacinthosmum
R. hellwigii
R. goodenoughii
R. alticola
R. caliginis
R. leptanthum
R. inconspicuum
R. lochae
R. commonae
R. herzogii
R. rappardii
R. flavoviride
R. konori var. konori
R. agathodaemonis
R. sp. nova
R. superbum
R. muscicola
R. rubineiflorum
R. gracilentum
R. anagalliflorum
R. vitis-idaea
R. loranthiflorum subsp. loranthiflorum
R. wentianum
R. wrightianum var. wrightianum
R. jasminiflorum subsp. jasminiflorum
R. orbiculatum
R. suaveolens
R. edanoi subsp. edanoi
R. ruttenii
R. radians
R. polyanthemum
R. stapfianum
R. burttii
R. abietifolium
R. rugosum var. rugosum
R. praetervisum
R. buxifolium
R. vidalii subsp. vidalii
R. taxifolium
R. rousei
R. longiflorum
R. citrinum
R. banghamiorum
R. javanicum subsp. kinabaluense
R. javanicum subsp. brookeanum 1
R. javanicum subsp. brookeanum 2
R. javanicum subsp. palawanense 1
R. javanicum subsp. palawanense 2
R. javanicum subsp. schadenbergii 2
R. javanicum subsp. schadenbergii 3
R. javanicum subsp. schadenbergii 1
R. cockburnii
R. javanicum subsp. cladotrichum 2
R. javanicum subsp. javanicum
R. multicolor
R. javanicum subsp. cladotrichum 1
R. robinsonii
R. rarilepidotum
R. javanicum subsp. gracile 1
R. javanicum subsp. gracile 2
R. acrophilum
R. retivenium 1
R. retivenium 2
R. javanicum subsp. moultonii
R. crassifolium
R. bagobonum 5
R. bagobonum 3
R. bagobonum 4
R. bagobonum 6
R. bagobonum 1
R. bagobonum 2
R. bagobonum 8
R. bagobonum 7
R. bagobonum 9
R. bagobonum 10
R. stenophyllum subsp. stenophyllum
R. lanceolatum
R. leptobrachion
R. celebicum
R. bloembergenii
R. impositum
R. kochii
R. lagunculicarpum
R. zollingeri
R. brassii
R. chevalieri
R. pauciflorum
R. album
R. lamrialianum subsp. lamrialianum
R. apoanum
R. malayanum
R. vinicolor
R. himantodes var. himantodes
R. pulleanum
R. ericoides
R. meliphagidum
R. quadrasianum var. quadrasianum
R. borneense subsp. villosum
R. retusum
R. scortechinii
R. perakense
R. kawakamii
R. rushforthii
R. emarginatum
R. vaccinioides
R. santapaui
R. minus
R. ferrugineum
R. anthopogon
R. primuliflorum
Clade 7
Clade 6
Clade 5b
Clade 4
Clade 5a
Clade 3
Clade 2
Clade 1

Fig. S8 SVDQuartets consensus topology of *Rhododendron* sect. *Schistante* that was generated from the min74 dataset, which required 74 samples per locus.
Support values at nodes for the ingroup are shown in the following order: Quartet Concordance/Quartet Differential/Quartet Informativeness/bootstrap. Seven main clades (1-7) were identified in the study and referred to based on subsectional classification: Pseudovireya, Discovireya, Malayovireya, Euvireya A, Euvireya B, Euvireya C, and Euvireya D, respectively.

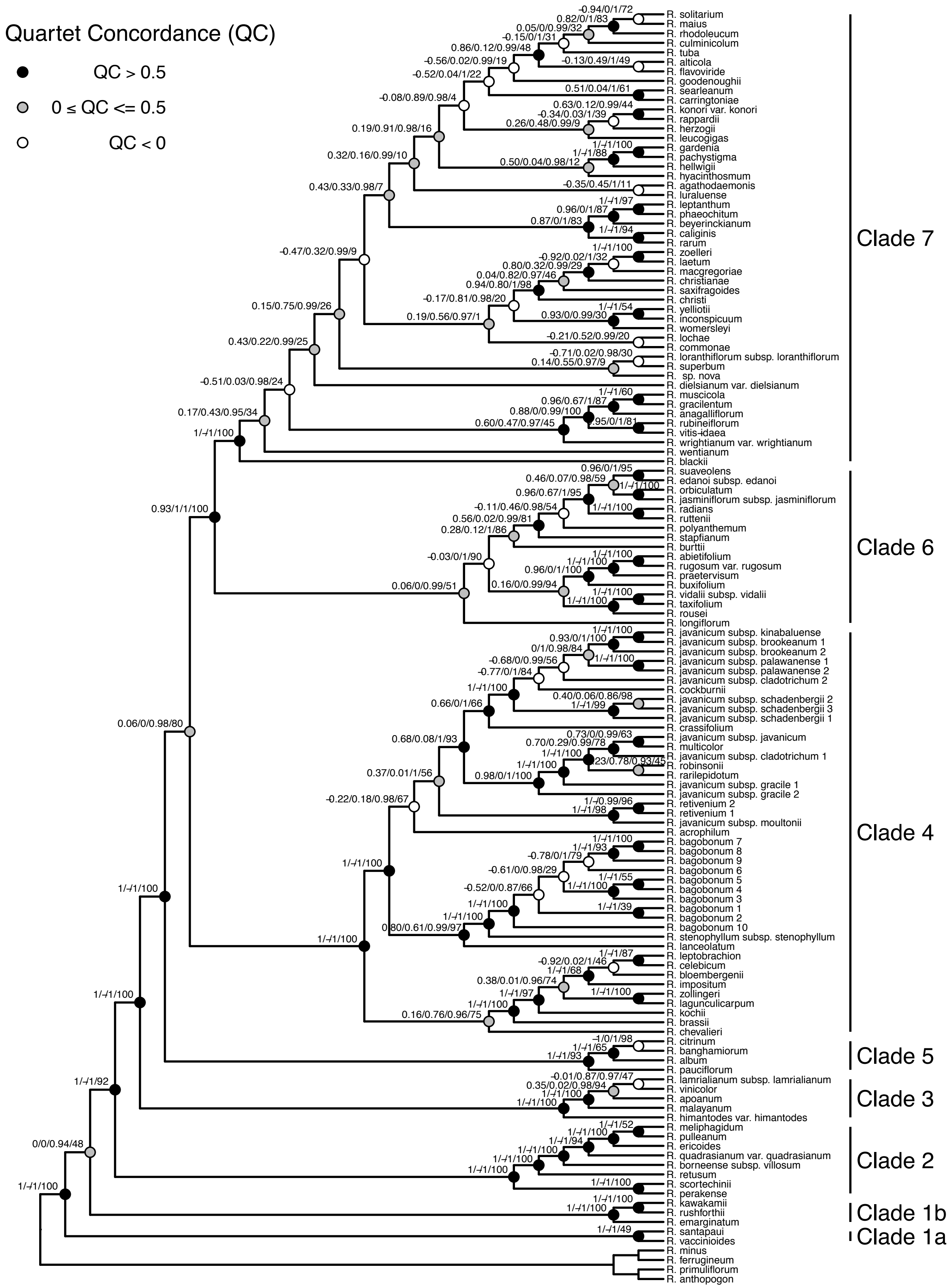
Quartet Concordance (QC)
QC > 0.5
0 ≤ QC <= 0.5
QC < 0
R. solitarium
R. maius
R. rhodoleucum
R. culminicolum
R. tuba
R. alticola
R. flavoviride
R. goodenoughii
R. searleanum
R. carringtoniae
R. konori var. konori
R. rappardii
R. herzogii
R. leucogigas
R. gardenia
R. pachystigma
R. hellwigii
R. hyacinthosmum
R. agathodaemonis
R. luraluense
R. leptanthum
R. phaeochitum
R. beyerinckianum
R. caliginis
R. rarum
R. zoelleri
R. laetum
R. macgregoriae
R. christianae
R. saxifragoides
R. christi
R. yelliotii
R. inconspicuum
R. womersleyi
R. lochae
R. commonae
R. loranthiflorum subsp. loranthiflorum
R. superbum
R. sp. nova
R. dielsianum var. dielsianum
R. muscicola
R. gracilentum
R. anagalliflorum
R. rubineiflorum
R. vitis-idaea
R. wrightianum var. wrightianum
R. wentianum
R. blackii
R. suaveolens
R. edanoi subsp. edanoi
R. orbiculatum
R. jasminiflorum subsp. jasminiflorum
R. radians
R. ruttenii
R. polyanthemum
R. stapfianum
R. burttii
R. abietifolium
R. rugosum var. rugosum
R. praetervisum
R. buxifolium
R. vidalii subsp. vidalii
R. taxifolium
R. rousei
R. longiflorum
R. javanicum subsp. kinabaluense
R. javanicum subsp. brookeanum 1
R. javanicum subsp. brookeanum 2
R. javanicum subsp. palawanense 1
R. javanicum subsp. palawanense 2
R. javanicum subsp. cladotrichum 2
R. cockburnii
R. javanicum subsp. schadenbergii 2
R. javanicum subsp. schadenbergii 3
R. javanicum subsp. schadenbergii 1
R. crassifolium
R. javanicum subsp. javanicum
R. multicolor
R. javanicum subsp. cladotrichum 1
R. robinsonii
R. rarilepidotum
R. javanicum subsp. gracile 1
R. javanicum subsp. gracile 2
R. retivenium 2
R. retivenium 1
R. javanicum subsp. moultonii
R. acrophilum
R. bagobonum 7
R. bagobonum 8
R. bagobonum 9
R. bagobonum 6
R. bagobonum 5
R. bagobonum 4
R. bagobonum 3
R. bagobonum 1
R. bagobonum 2
R. bagobonum 10
R. stenophyllum subsp. stenophyllum
R. lanceolatum
R. leptobrachion
R. celebicum
R. bloembergenii
R. impositum
R. zollingeri
R. lagunculicarpum
R. kochii
R. brassii
R. chevalieri
R. citrinum
R. banghamiorum
R. album
R. pauciflorum
R. lamrialianum subsp. lamrialianum
R. vinicolor
R. apoanum
R. malayanum
R. himantodes var. himantodes
R. meliphagidum
R. pulleanum
R. ericoides
R. quadrasianum var. quadrasianum
R. borneense subsp. villosum
R. retusum
R. scortechinii
R. perakense
R. kawakamii
R. rushforthii
R. emarginatum
R. santapaui
R. vaccinioides
R. minus
R. ferrugineum
R. primuliflorum
R. anthopogon
Clade 7
Clade 6
Clade 4
Clade 5
Clade 3
Clade 2
Clade 1b
Clade 1a

Fig. S9 SVDQuartets consensus topology of *Rhododendron* sect. *Schistante* that was generated from the min111 dataset, which required 111 samples per locus.
Support values at nodes for the ingroup are shown in the following order: Quartet Concordance/Quartet Differential/Quartet Informativeness/bootstrap. Seven main clades (1-7) were identified in the study and referred to based on subsectional classification: Pseudovireya, Discovireya, Malayovireya, Euvireya A, Euvireya B, Euvireya C, and Euvireya D, respectively.

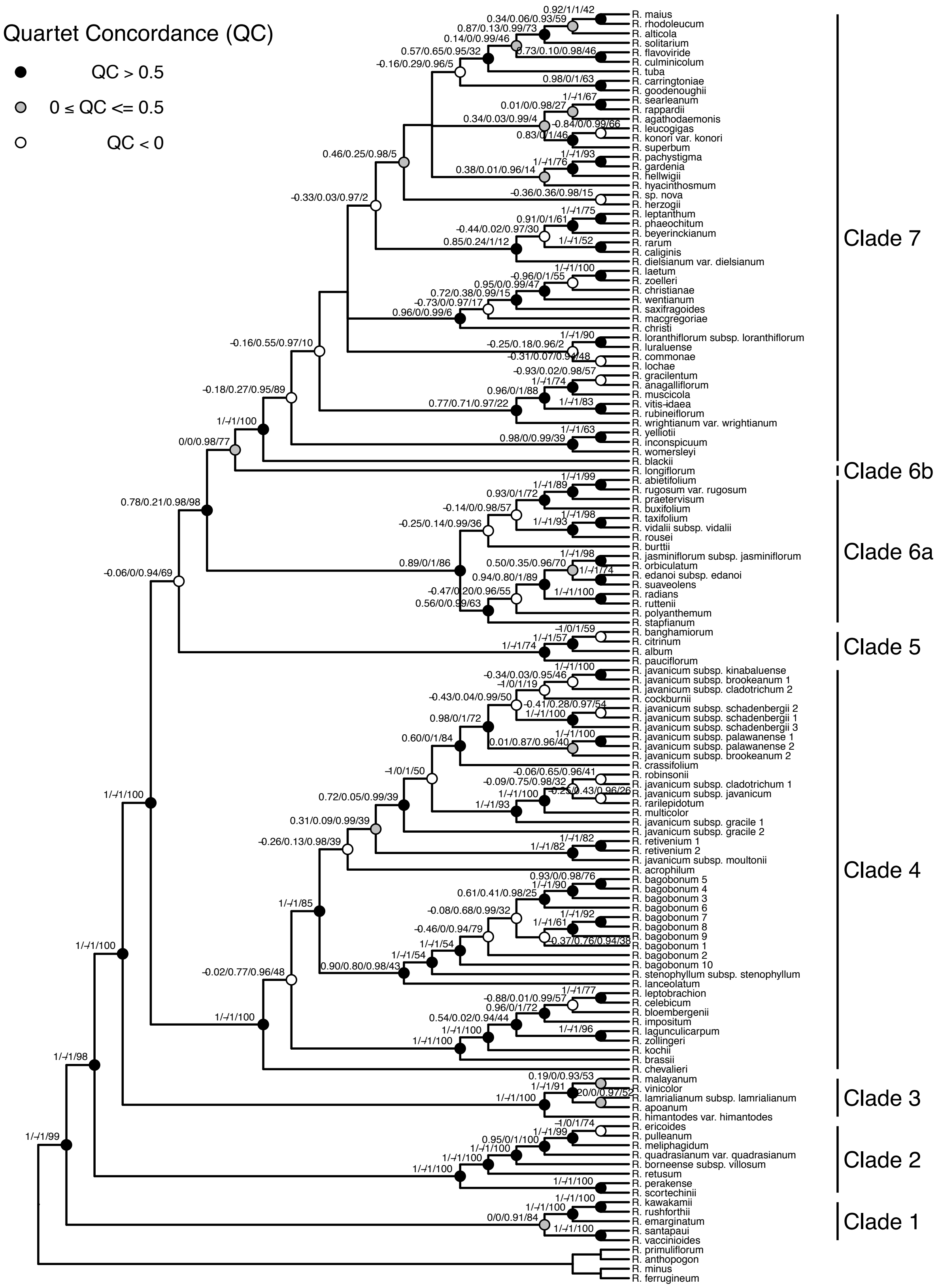

Quartet Concordance (QC)
QC > 0.5
0 ≤ QC <= 0.5
QC < 0
R. maius
R. rhodoleucum
R. alticola
R. solitarium
R. flavoviride
R. culminicolum
R. tuba
R. carringtoniae
R. goodenoughii
R. searleanum
R. rappardii
R. agathodaemonis
R. leucogigas
R. konori var. konori
R. superbum
R. pachystigma
R. gardenia
R. hellwigii
R. hyacinthosmum
R. sp. nova
R. herzogii
R. leptanthum
R. phaeochitum
R. beyerinckianum
R. rarum
R. caliginis
R. dielsianum var. dielsianum
R. laetum
R. zoelleri
R. christianae
R. wentianum
R. saxifragoides
R. macgregoriae
R. christi
R. loranthiflorum subsp. loranthiflorum
R. luraluense
R. commonae
R. lochae
R. gracilentum
R. anagalliflorum
R. muscicola
R. vitis-idaea
R. rubineiflorum
R. wrightianum var. wrightianum
R. yelliotii
R. inconspicuum
R. womersleyi
R. blackii
R. longiflorum
R. abietifolium
R. rugosum var. rugosum
R. praetervisum
R. buxifolium
R. taxifolium
R. vidalii subsp. vidalii
R. rousei
R. burttii
R. jasminiflorum subsp. jasminiflorum
R. orbiculatum
R. edanoi subsp. edanoi
R. suaveolens
R. radians
R. ruttenii
R. polyanthemum
R. stapfianum
R. banghamiorum
R. citrinum
R. album
R. pauciflorum
R. javanicum subsp. kinabaluense
R. javanicum subsp. brookeanum 1
R. javanicum subsp. cladotrichum 2
R. cockburnii
R. javanicum subsp. schadenbergii 2
R. javanicum subsp. schadenbergii 1
R. javanicum subsp. schadenbergii 3
R. javanicum subsp. palawanense 1
R. javanicum subsp. palawanense 2
R. javanicum subsp. brookeanum 2
R. crassifolium
R. robinsonii
R. javanicum subsp. cladotrichum 1
R. javanicum subsp. javanicum
R. rarilepidotum
R. multicolor
R. javanicum subsp. gracile 1
R. javanicum subsp. gracile 2
R. retivenium 1
R. retivenium 2
R. javanicum subsp. moultonii
R. acrophilum
R. bagobonum 5
R. bagobonum 4
R. bagobonum 3
R. bagobonum 6
R. bagobonum 7
R. bagobonum 8
R. bagobonum 9
R. bagobonum 1
R. bagobonum 2
R. bagobonum 10
R. stenophyllum subsp. stenophyllum
R. lanceolatum
R. leptobrachion
R. celebicum
R. bloembergenii
R. impositum
R. lagunculicarpum
R. zollingeri
R. kochii
R. brassii
R. chevalieri
R. malayanum
R. vinicolor
R. lamrialianum subsp. lamrialianum
R. apoanum
R. himantodes var. himantodes
R. ericoides
R. pulleanum
R. meliphagidum
R. quadrasianum var. quadrasianum
R. borneense subsp. villosum
R. retusum
R. perakense
R. scortechinii
R. kawakamii
R. rushforthii
R. emarginatum
R. santapaui
R. vaccinioides
R. primuliflorum
R. anthopogon
R. minus
R. ferrugineum
Clade 7
Clade 6b
Clade 6a
Clade 5
Clade 4
Clade 3
Clade 2
Clade 1

Fig. S10 Heatmap of D_{min}-statistics in *Rhododendron* sect. *Schistanthe.*
D-statistics were calculated for all possible trios without assuming any knowledge of relationships; then, the lowest *D*-statistic for each trio was used as a conservative estimate. P2 and P3 taxa are arranged along the vertical and horizontal axes. Clade designations and numbers are indicated along taxon names: Pse = Pseudovireya, Dis = Discovireya, Mal = Malayovireya, EuA = Euvireya A, EuB = Euvireya B, EuC = Euvireya C, and EuD = Euvireya D. The most significant *D*-statistic found for two species across all possible P1 taxa is indicated by heatmap cells. Red cells indicate higher *D*-statistics (presence of introgression), blue cells indicate lower *D*-statistics (absence of introgression), increasing color saturation indicates greater significance based on *P*-values, and white cells indicate lack of information.

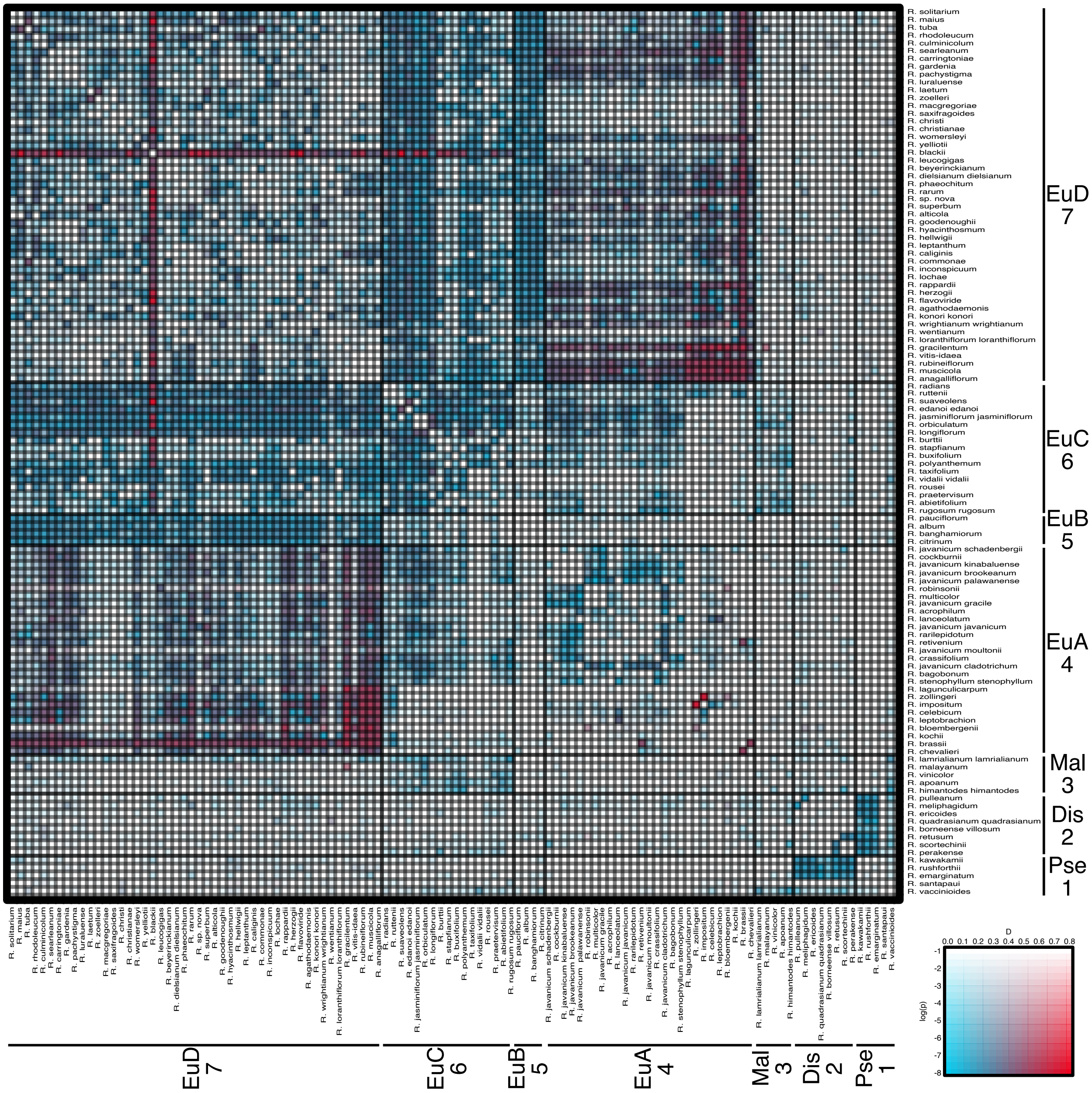

Fig. S11 Heatmap of D_{tree}-statistics in *Rhododendron* sect. *Schistanthe.*
D-statistics were calculated using the species relationships from the SVDQuartets analyses of the min4 dataset. P2 and P3 taxa are arranged along the vertical and horizontal axes. Clade designations and numbers are indicated along taxon names: Pse = Pseudovireya, Dis = Discovireya, Mal = Malayovireya, EuA = Euvireya A, EuB = Euvireya B, EuC = Euvireya C, and EuD = Euvireya D. The most significant *D*-statistic found for two species across all possible P1 taxa is indicated by heatmap cells. Red cells indicate higher *D*-statistics (presence of introgression), blue cells indicate lower *D*-statistics (absence of introgression), increasing color saturation indicates greater significance based on *P*-values, and white cells indicate lack of information.

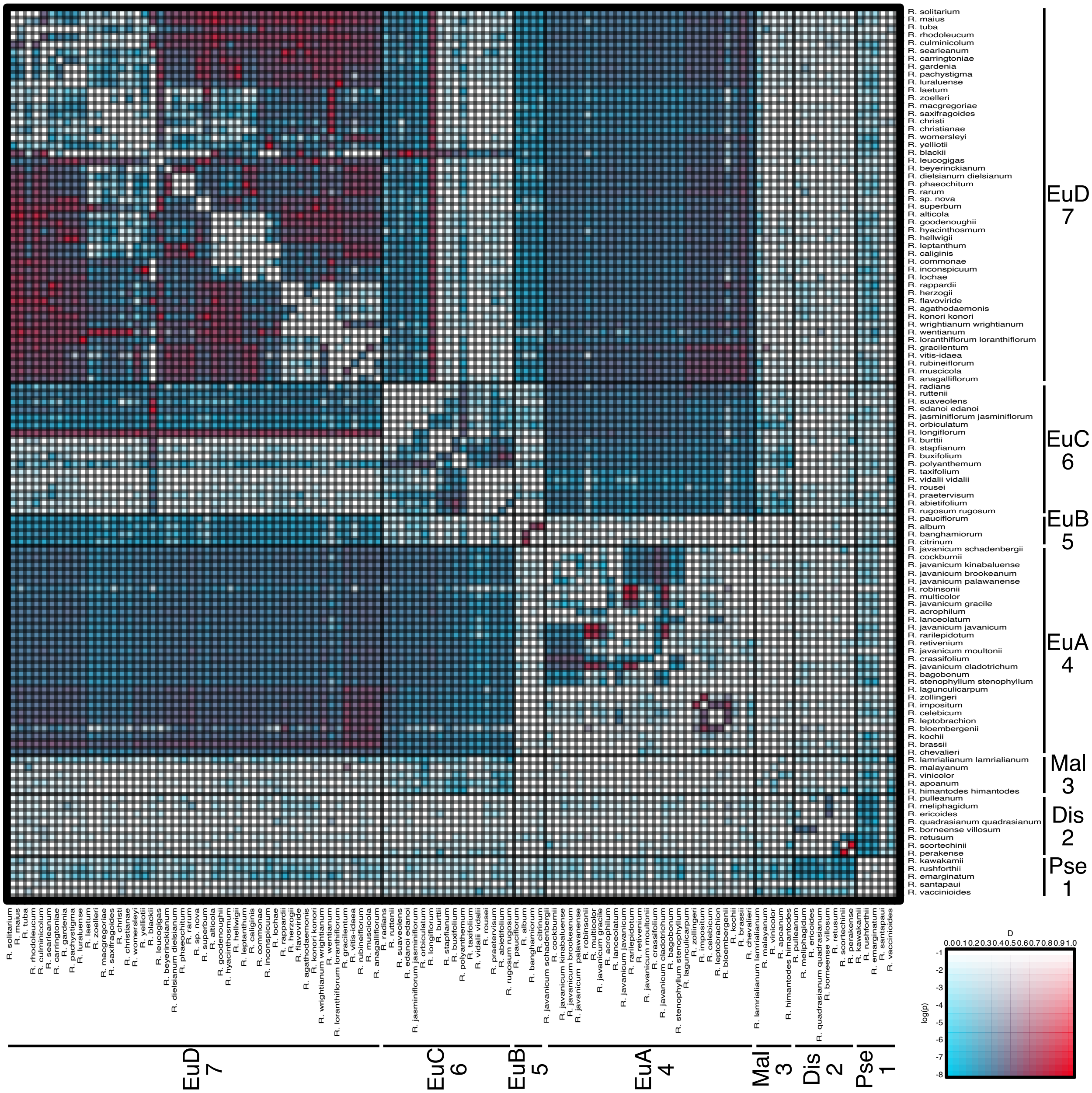

Fig. S12 Panel of outlines used to reconstruct floral morphospaces for *Rhododendron* sect. *Schistanthe*.
Specific epithets corresponding to each outline are indicated inside each outline. (a) Front-view outlines of corollas. (b) Side-view outlines of corollas.

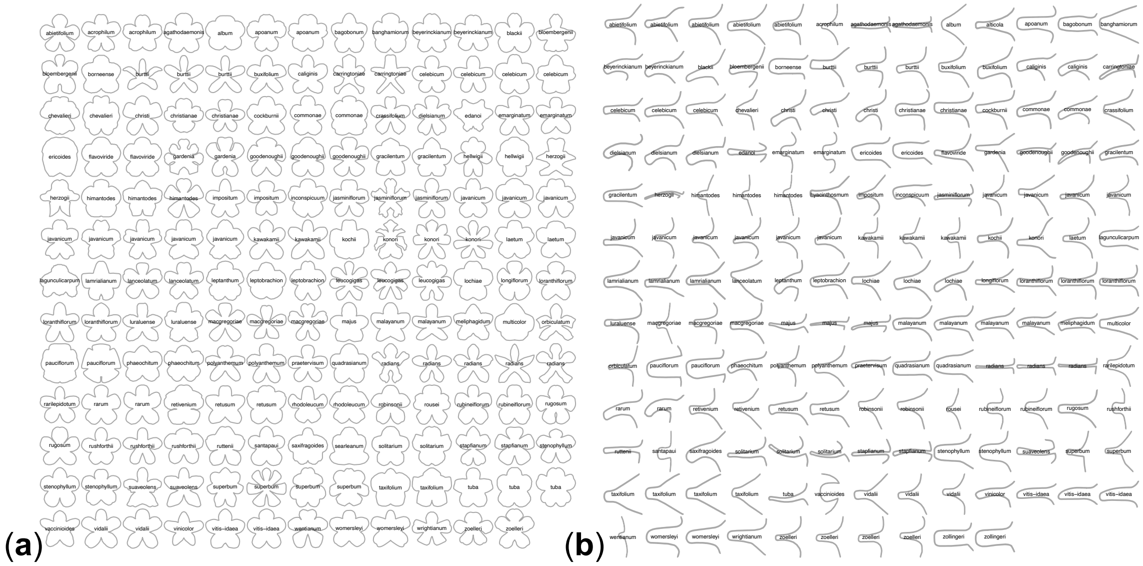

Fig. S13 Morphospace variation and clustering of corollas from *Rhododendron* sect. *Schistanthe* using principal components analysis (PCA).

Colored dots and polygons correspond to the number of clusters identified by K-means clustering. Polygons represent total morphospace occupied by each cluster. (a-b) Morphospace for the front view of corollas (K = 5). (c-d) Morphospace for the side view of corollas (K = 2).

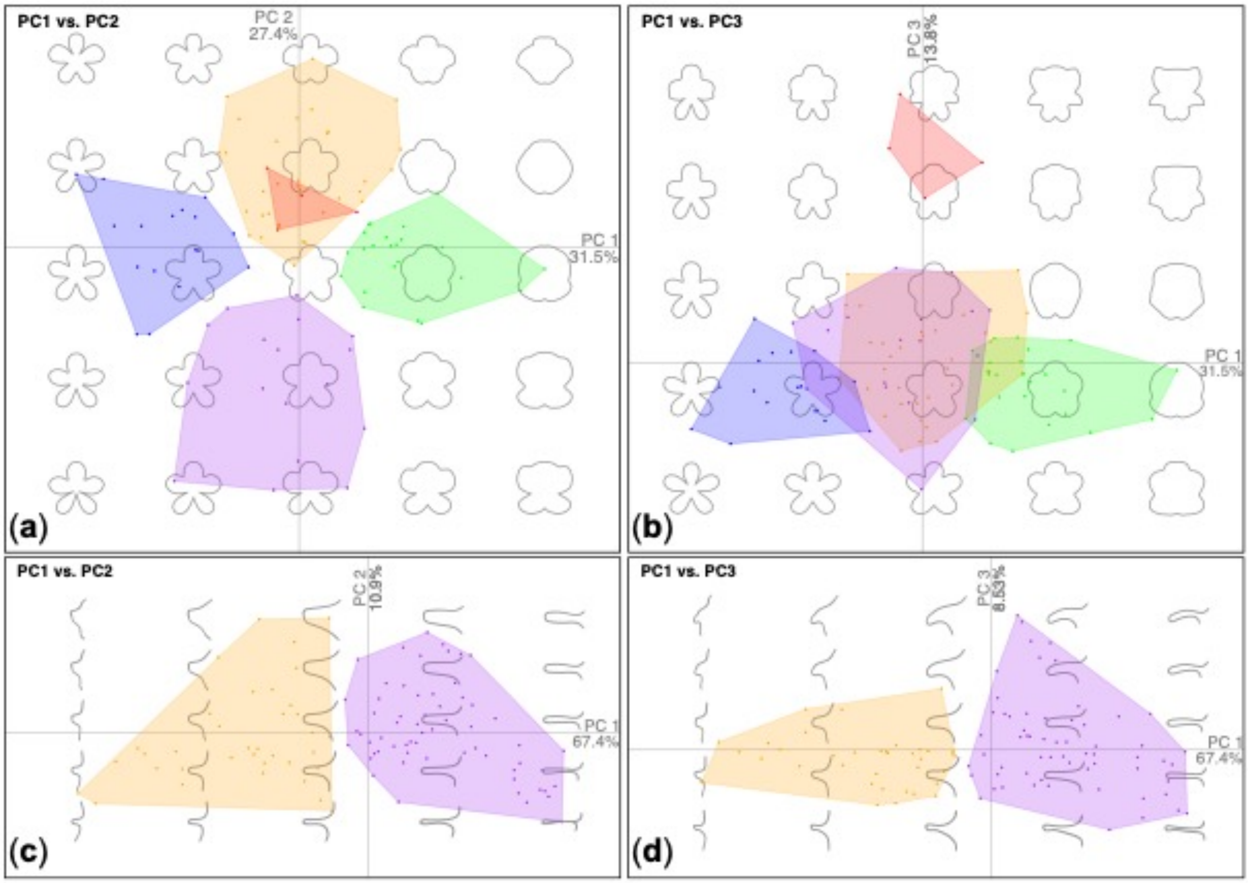

Fig. S14 Morphospace variation of corollas from *Rhododendron* sect. *Schistanthe* using principal components analysis (PCA) and flower color.

Colored dots and polygons correspond to flower color in (b). Polygons represent total morphospace occupied by each flower color. (a-b) Morphospace for the front view of corollas. (c-d) Morphospace for the side view of corollas.

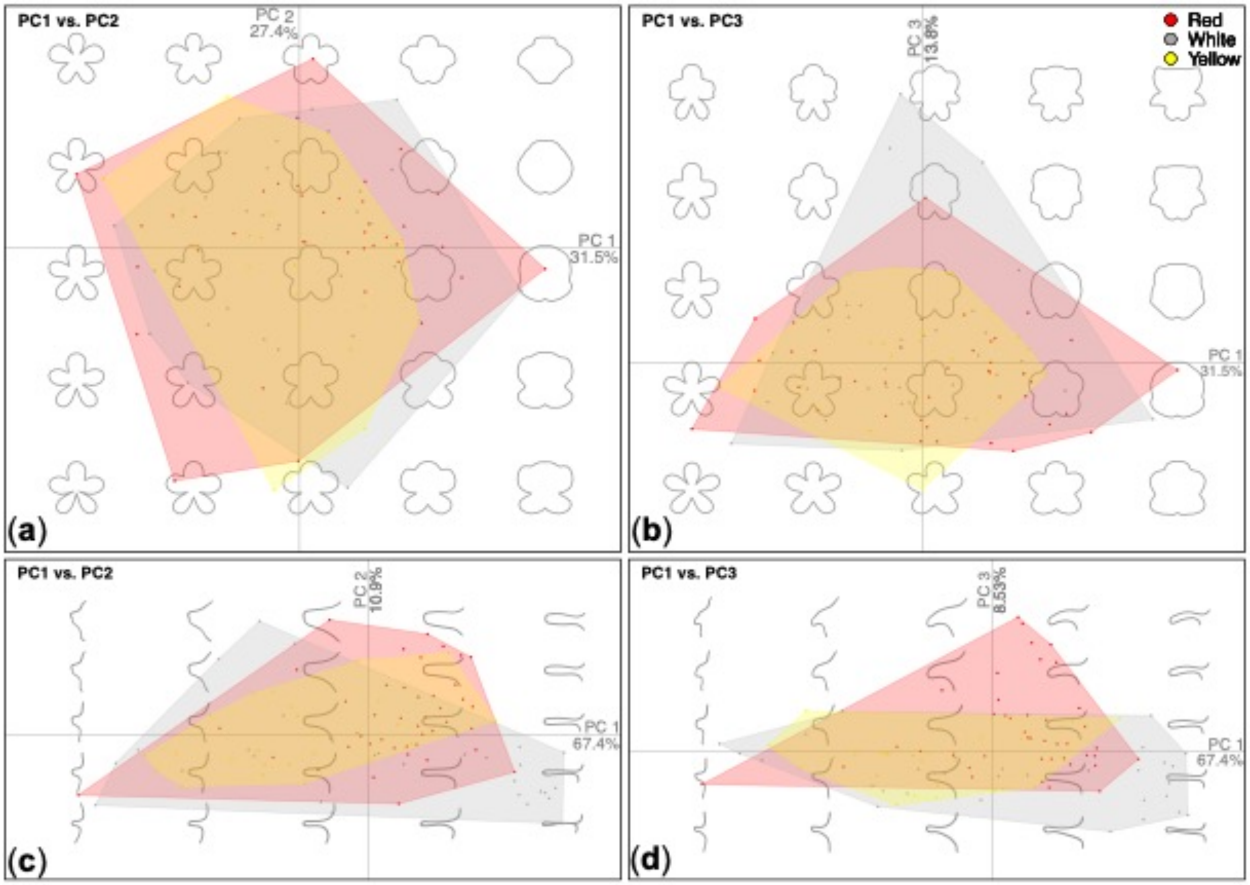

Table S1 Samples and vouchers used in this study.

Taxon	Collection locality	Voucher information
Empetrum nigrum L.	Cultivated, USA, Washington, Seattle, University of Washington, Medicinal Herb Garden	Soza 1957 (WTU)
Erica arborea L.	Cultivated, USA, Washington, Seattle, University of Washington Botanic Gardens, Washington Park Arboretum, Accession No. 184-81	Barczak 34 (WTUH)
Erica scoparia L.	Cultivated, USA, Washington, Seattle, University of Washington Botanic Gardens, Washington Park Arboretum, Accession No. 303-81	N/A[1]
Kalmia latifolia L.	Cultivated, USA, Washington, Seattle, University of Washington Botanic Gardens, Washington Park Arboretum, Accession No. 1054-40	Herbarium Committee 1935 (WTUH)
Kalmia sp.	Cultivated, USA, Washington, Seattle	N/A
Kalmiopsis fragrans Meinke & Kaye	Cultivated, USA, Washington, Seattle, University of Washington Botanic Gardens, Washington Park Arboretum, Accession No. 106-13	Rickenbaker 125 (WTUH)
Rhododendron abietifolium Sleumer	Cultivated, UK, Scotland, Royal Botanic Garden Edinburgh, Accession No. 19801209	Conlon C216 (E)
Rhododendron acrophilum Merr. & Quisumb.	Cultivated, UK, Scotland, Royal Botanic Garden Edinburgh, Accession No. 19922768	Conlon C424 (E)
Rhododendron agathodaemonis J.J.Sm.	Cultivated, UK, Scotland, Royal Botanic Garden Edinburgh, Accession No. 20080989	N/A
Rhododendron albiflorum Hook.	Cultivated, USA, Washington, Federal Way, Rhododendron	N/A

	Species Botanical Garden, Accession No. 2001/117	
Rhododendron albrechtii Maxim	Cultivated, USA, Washington, Federal Way, Rhododendron Species Botanical Garden, Accession No. 1999/105	Soza & Ramage 1953 (WTU)
Rhododendron album Blume	Cultivated, UK, Scotland, Royal Botanic Garden Edinburgh, Accession No. 19882540	Conlon C127 (E)
Rhododendron alticola Sleumer	Cultivated, UK, Scotland, Royal Botanic Garden Edinburgh, Accession No. 19710182	Conlon C59 (E)
Rhododendron anagalliflorum Wernham	Cultivated, UK, Scotland, Royal Botanic Garden Edinburgh, Accession No. 19821953	Conlon C497 (E)
Rhododendron anthopogon D. Don	Cultivated, USA, Washington, Federal Way, Rhododendron Species Botanical Garden, Accession No. 1966/588	Goetsch 036 (WTU)
Rhododendron apoanum Stein	Cultivated, UK, Scotland, Royal Botanic Garden Edinburgh, Accession No. 19933034	Conlon C261 (E)
Rhododendron bagobonum H.F.Copel. #9	Cultivated, UK, Scotland, Royal Botanic Garden Edinburgh, Accession No. 19820933	Galloway 8 (E)
Rhododendron bagobonum H.F.Copel. #5	Cultivated, UK, Scotland, Royal Botanic Garden Edinburgh, Accession No. 20040442	Galloway 9 (E)
Rhododendron bagobonum H.F.Copel. #6	Cultivated, UK, Scotland, Royal Botanic Garden Edinburgh, Accession No. 19991922	Argent *s.n.* (E)
Rhododendron bagobonum H.F.Copel. #10	Cultivated, UK, Scotland, Royal Botanic Garden Edinburgh, Accession No. 19982490	Galloway 10 (E)
Rhododendron bagobonum	Cultivated, UK, Scotland,	Galloway 11 (E)

H.F.Copel. #2	Royal Botanic Garden Edinburgh, Accession No. 19821504	
Rhododendron bagobonum H.F.Copel. #7	Cultivated, UK, Scotland, Royal Botanic Garden Edinburgh, Accession No. 19801146	Galloway 12 (E)
Rhododendron bagobonum H.F.Copel. #3	Cultivated, UK, Scotland, Royal Botanic Garden Edinburgh, Accession No. 20000482	Galloway 13 (E)
Rhododendron bagobonum H.F.Copel. #8	Cultivated, UK, Scotland, Royal Botanic Garden Edinburgh, Accession No. 19922741	Galloway 14 (E)
Rhododendron bagobonum H.F.Copel. #1	Cultivated, UK, Scotland, Royal Botanic Garden Edinburgh, Accession No. 19922777	Argent *s.n.* (E)
Rhododendron bagobonum H.F.Copel. #4	Cultivated, UK, Scotland, Royal Botanic Garden Edinburgh, Accession No. 20080074	Conlon C396 (E)
Rhododendron banghamiorum (J.J.Sm.) Sleumer	Cultivated, UK, Scotland, Royal Botanic Garden Edinburgh, Accession No. 20010334	Conlon C487 (E)
Rhododendron beyerinckianum Koord.	Cultivated, USA, Washington, Federal Way, Rhododendron Species Botanical Garden, Accession No. 1985/047	Soza *et al.* 1926/1933 (WTU)
Rhododendron blackii Sleumer	Cultivated, USA, Washington, Federal Way, Rhododendron Species Botanical Garden, Accession No. 2017/033	Soza *et al.* 1927 (WTU)
Rhododendron bloembergenii Sleumer	Cultivated, UK, Scotland, Royal Botanic Garden Edinburgh, Accession No. 19973620	Argent *s.n.* (E)
Rhododendron borneense (J.J.Sm.) Argent, A.L.Lamb & Phillipps subsp. *villosum* (J.J.Sm.) Argent, A.L.Lamb &	Cultivated, UK, Scotland, Royal Botanic Garden Edinburgh, Accession No. 19821510	Conlon C461 (E)

Phillipps		
Rhododendron brassii Sleumer	Cultivated, UK, Scotland, Royal Botanic Garden Edinburgh, Accession No. 19930917	Conlon C477 (E)
Rhododendron burttii P.Woods	Cultivated, UK, Scotland, Royal Botanic Garden Edinburgh, Accession No. 19672565	Conlon C50 (E)
Rhododendron buxifolium H.Low ex Hook.f.	Cultivated, UK, Scotland, Royal Botanic Garden Edinburgh, Accession No. 19962012	Conlon C405 (E)
Rhododendron caliginis Kores	Cultivated, USA, Washington, Federal Way, Rhododendron Species Botanical Garden, Accession No. 1989/003	Soza *et al.* 1928 (WTU)
Rhododendron camtschaticum Pall.	Cultivated, USA, Washington, Federal Way, Rhododendron Species Botanical Garden, Accession No. 1977/080	Soza & Ramage 1934 (WTU)
Rhododendron carringtoniae F.Muell.	Cultivated, UK, Scotland, Royal Botanic Garden Edinburgh, Accession No. 19741502	Conlon C240 (E)
Rhododendron celebicum DC.	Cultivated, USA, Washington, Federal Way, Rhododendron Species Botanical Garden, Accession No. 1997/062	Soza 1964 (WTU)
Rhododendron chevalieri Dop ex A.Chev.	Cultivated, UK, Scotland, Royal Botanic Garden Edinburgh, Accession No. 20010651	Conlon C285 (E)
Rhododendron christi F.Foerst.	Cultivated, UK, Scotland, Royal Botanic Garden Edinburgh, Accession No. 19861623	Conlon C356 (E)
Rhododendron christianae Sleumer	Cultivated, UK, Scotland, Royal Botanic Garden Edinburgh, Accession No. 19731628	Conlon C28 (E)
Rhododendron citrinum Hassk.	Cultivated, UK, Scotland, Royal Botanic Garden	Conlon C135 (E)

	Edinburgh, Accession No. 19842322	
Rhododendron cockburnii (Argent, A.L.Lamb & Phillipps) Craven	Cultivated, UK, Scotland, Royal Botanic Garden Edinburgh, Accession No. 20080655	Conlon C431 (E)
Rhododendron commonae F.Foerst.	Cultivated, UK, Scotland, Royal Botanic Garden Edinburgh, Accession No. 19973645	Conlon C468 (E)
Rhododendron crassifolium Stapf	Cultivated, UK, Scotland, Royal Botanic Garden Edinburgh, Accession No. 19801206	Conlon C218 (E)
Rhododendron culminicolum F.Muell.	Cultivated, USA, Washington, Federal Way, Rhododendron Species Botanical Garden, SEH #11023	N/A
Rhododendron dielsianum Schltr. var. *dielsianum*	Cultivated, UK, Scotland, Royal Botanic Garden Edinburgh, Accession No. 19752618	N/A
Rhododendron edanoi Merr. & Quisumb. subsp. *edanoi*	Cultivated, UK, Scotland, Royal Botanic Garden Edinburgh, Accession No. 19981819	Conlon C44 (E)
Rhododendron emarginatum Hemsl. & E.H.Wilson	Cultivated, USA, Washington, Federal Way, Rhododendron Species Botanical Garden, Accession No. 1996/054	N/A
Rhododendron ericoides H.Low ex Hook.f.	Cultivated, UK, Scotland, Royal Botanic Garden Edinburgh, Accession No. 19871806	Conlon C21 (E)
Rhododendron ferrugineum L.	Cultivated, USA, Washington, Federal Way, Rhododendron Species Botanical Garden, Accession No. 1998/772	Goetsch 045 (WTU)
Rhododendron flavoviride J.J.Sm.	Cultivated, UK, Scotland, Royal Botanic Garden Edinburgh, Accession No. 19930971	Conlon C313 (E)
Rhododendron gardenia	Cultivated, UK, Scotland,	Conlon C320 (E)

Schltr.	Royal Botanic Garden Edinburgh, Accession No. 19650265	
Rhododendron goodenoughii Sleumer	Cultivated, UK, Scotland, Royal Botanic Garden Edinburgh, Accession No. 19772400	Conlon C208 (E)
Rhododendron gracilentum F.Muell.	Cultivated, USA, Washington, Federal Way, Rhododendron Species Botanical Garden, Accession No. 1978/103	Goetsch 104 (WTU)
Rhododendron hellwigii Warb.	Cultivated, UK, Scotland, Royal Botanic Garden Edinburgh, Accession No. 19762978	Conlon C379 (E)
Rhododendron herzogii Warb.	Cultivated, USA, Washington, Federal Way, Rhododendron Species Botanical Garden, Accession No. 1989/004	Goetsch 068 (WTU)
Rhododendron himantodes Sleumer var. *himantodes*	Cultivated, USA, Washington, Federal Way, Rhododendron Species Botanical Garden, Accession No. 2007/020	Soza 1963 (WTU)
Rhododendron hyacinthosmum Sleumer	Cultivated, UK, Scotland, Royal Botanic Garden Edinburgh, Accession No. 19750104	Conlon C211 (E)
Rhododendron impositum J.J.Sm.	Cultivated, USA, Washington, Federal Way, Rhododendron Species Botanical Garden, Accession No. 1997/055	Soza 1961 (WTU)
Rhododendron inconspicuum J.J.Sm.	Cultivated, UK, Scotland, Royal Botanic Garden Edinburgh, Accession No. 19614237	Conlon C129 (E)
Rhododendron jasminiflorum Hook. subsp. *jasminiflorum*	Cultivated, UK, Scotland, Royal Botanic Garden Edinburgh, Accession No. 19680638	Conlon C113 (E)
Rhododendron javanicum Benn. subsp. *brookeanum* (H.Low ex Lindl.) Argent & Phillipps #2	Cultivated, UK, Scotland, Royal Botanic Garden Edinburgh, Accession No. 19841173	Galloway 15 (E)

Rhododendron javanicum Benn. subsp. *brookeanum* (H.Low ex Lindl.) Argent & Phillipps #1	Cultivated, UK, Scotland, Royal Botanic Garden Edinburgh, Accession No. 19773440	Galloway 16 (E)
Rhododendron javanicum Benn. subsp. *cladotrichum* (Sleumer) Argent #2	Cultivated, UK, Scotland, Royal Botanic Garden Edinburgh, Accession No. 19952758	Conlon C99 (E)
Rhododendron javanicum Benn. subsp. *cladotrichum* (Sleumer) Argent #1	Cultivated, UK, Scotland, Royal Botanic Garden Edinburgh, Accession No. 19913084	Argent *s.n.* (E)
Rhododendron javanicum Benn. subsp. *gracile* (Lindl.) Argent, A.L.Lamb & Phillipps #1	Cultivated, UK, Scotland, Royal Botanic Garden Edinburgh, Accession No. 19831219	Galloway 17 (E)
Rhododendron javanicum Benn. subsp. *gracile* (Lindl.) Argent, A.L.Lamb & Phillipps #2	Cultivated, UK, Scotland, Royal Botanic Garden Edinburgh, Accession No. 19851901	Conlon C387 (E)
Rhododendron javanicum Benn. subsp. *javanicum*	Cultivated, UK, Scotland, Royal Botanic Garden Edinburgh, Accession No. 19812954	Conlon C60 (E)
Rhododendron javanicum Benn. subsp. *kinabaluense* (Argent, A.L.Lamb & Phillipps) Argent	Cultivated, UK, Scotland, Royal Botanic Garden Edinburgh, Accession No. 19690955	Conlon C365 (E)
Rhododendron javanicum Benn. subsp. *moultonii* (Ridl.) Argent	Cultivated, UK, Scotland, Royal Botanic Garden Edinburgh, Accession No. 19913192	Conlon C159 (E)
Rhododendron javanicum Benn. subsp. *palawanense* Argent #1	Cultivated, UK, Scotland, Royal Botanic Garden Edinburgh, Accession No. 19922762	Conlon C24 (E)
Rhododendron javanicum Benn. subsp. *palawanense* Argent #2	Cultivated, UK, Scotland, Royal Botanic Garden Edinburgh, Accession No. 19981796	N/A
Rhododendron javanicum Benn. subsp. *schadenbergii* (Warb.) Argent #2	Cultivated, UK, Scotland, Royal Botanic Garden Edinburgh, Accession No.	Argent *s.n.* (E)

	19991884	
Rhododendron javanicum Benn. subsp. *schadenbergii* (Warb.) Argent #1	Cultivated, UK, Scotland, Royal Botanic Garden Edinburgh, Accession No. 19922809	Conlon C309 (E)
Rhododendron javanicum Benn. subsp. *schadenbergii* (Warb.) Argent #3	Cultivated, UK, Scotland, Royal Botanic Garden Edinburgh, Accession No. 19991944	N/A
Rhododendron kawakamii Hayata	Cultivated, UK, Scotland, Royal Botanic Garden Edinburgh, Accession No. 19902930	N/A
Rhododendron kochii Stein	Cultivated, UK, Scotland, Royal Botanic Garden Edinburgh, Accession No. 19972500	Conlon C198 (E)
Rhododendron konori Becc. var. *konori*	Cultivated, USA, Washington, Federal Way, Rhododendron Species Botanical Garden, Accession No. 1979/036	Goetsch 069 (WTU)
Rhododendron laetum J.J.Sm	Cultivated, UK, Scotland, Royal Botanic Garden Edinburgh, Accession No. 19670214	Conlon C70 (E)
Rhododendron lagunculicarpum J.J.Sm.	Cultivated, UK, Scotland, Royal Botanic Garden Edinburgh, Accession No. 19981654	Conlon C201 (E)
Rhododendron lamrialianum Argent & T.J.Barkman subsp. *lamrialianum*	Cultivated, UK, Scotland, Royal Botanic Garden Edinburgh, Accession No. 19841164	Conlon C94 (E)
Rhododendron lanceolatum Ridl.	Cultivated, UK, Scotland, Royal Botanic Garden Edinburgh, Accession No. 20010617	Conlon C168 (E)
Rhododendron leptanthum F.Muell.	Cultivated, UK, Scotland, Royal Botanic Garden Edinburgh, Accession No. 19681436	Conlon C409 (E)
Rhododendron leptobrachion Sleumer	Cultivated, USA, Washington, Federal Way, Rhododendron	Soza 1959 (WTU)

	Species Botanical Garden, Accession No. 2017/059	
Rhododendron leucogigas Sleumer	Cultivated, UK, Scotland, Royal Botanic Garden Edinburgh, Accession No. 19682431	Conlon C138 (E)
Rhododendron lochae F.Muell.	Cultivated, UK, Scotland, Royal Botanic Garden Edinburgh, Accession No. 19961303	Argent *s.n.* (E)
Rhododendron longiflorum Lindl.	Cultivated, UK, Scotland, Royal Botanic Garden Edinburgh, Accession No. 19821503	Conlon C478 (E)
Rhododendron loranthiflorum Sleumer subsp. *loranthiflorum* 'Dick Shaw'	Cultivated, UK, Scotland, Royal Botanic Garden Edinburgh, Accession No. 19830533	Galloway 1 (E)
Rhododendron luraluense Sleumer	Cultivated, UK, Scotland, Royal Botanic Garden Edinburgh, Accession No. 19830534	Conlon C436 (E)
Rhododendron macgregoriae F.Muell.	Cultivated, UK, Scotland, Royal Botanic Garden Edinburgh, Accession No. 19681160	Conlon C297 (E)
Rhododendron macrophyllum D. Don ex G. Don	USA, Oregon, Whitewater Road NF-2243	N/A
Rhododendron maius (J.J.Sm.) Sleumer	Cultivated, UK, Scotland, Royal Botanic Garden Edinburgh, Accession No. 19681423	Conlon C294 (E)
Rhododendron malayanum Jack	Cultivated, UK, Scotland, Royal Botanic Garden Edinburgh, Accession No. 19820746	Galloway 18 (E)
Rhododendron meliphagidum J.J.Sm.	Cultivated, UK, Scotland, Royal Botanic Garden Edinburgh, Accession No. 19880517	Conlon C114 (E)
Rhododendron minus Michx.	Cultivated, USA, Washington, Federal Way, Rhododendron Species Botanical Garden,	Goetsch 054 (WTU)

	Accession No. 1998/173	
Rhododendron molle G.Don	Cultivated, USA, Washington, Federal Way, Rhododendron Species Botanical Garden, Accession No. 1980/091	Soza & Ramage 1932 (WTU)
Rhododendron multicolor Miq.	Cultivated, UK, Scotland, Royal Botanic Garden Edinburgh, Accession No. 20110585	N/A
Rhododendron muscicola J.J.Sm.	Cultivated, UK, Scotland, Royal Botanic Garden Edinburgh, Accession No. 19943025	N/A
Rhododendron orbiculatum Ridl.	Cultivated, UK, Scotland, Royal Botanic Garden Edinburgh, Accession No. 19982489	Conlon C361 (E)
Rhododendron pachystigma Sleumer	Cultivated, UK, Scotland, Royal Botanic Garden Edinburgh, Accession No. 20061685	N/A
Rhododendron pauciflorum King & Gamble	Cultivated, UK, Scotland, Royal Botanic Garden Edinburgh, Accession No. 19750119	Conlon C18 (E)
Rhododendron perakense King & Gamble	Cultivated, UK, Scotland, Royal Botanic Garden Edinburgh, Accession No. 19973642	Conlon C231 (E)
Rhododendron periclymenoides (Michx.) Shinners	Cultivated, USA, Washington, Federal Way, Rhododendron Species Botanical Garden, Accession No. 1976/292	N/A
Rhododendron phaeochitum F.Muell.	Cultivated, USA, Washington, Federal Way, Rhododendron Species Botanical Garden, Accession No. 1986/022	Soza 1960 (WTU)
Rhododendron polyanthemum Sleumer	Cultivated, UK, Scotland, Royal Botanic Garden Edinburgh, Accession No. 19801295	Conlon C305 (E)
Rhododendron praetervisum Sleumer	Cultivated, UK, Scotland, Royal Botanic Garden	Conlon C89 (E)

	Edinburgh, Accession No. 19790954	
Rhododendron primuliflorum Bureau & Franch.	Cultivated, USA, Washington, Federal Way, Rhododendron Species Botanical Garden, Accession No. 1977/603	N/A
Rhododendron pulleanum Koord.	Cultivated, UK, Scotland, Royal Botanic Garden Edinburgh, Accession No. 20002004	N/A
Rhododendron quadrasianum S.Vidal var. *quadrasianum*	Cultivated, UK, Scotland, Royal Botanic Garden Edinburgh, Accession No. 19972518	Conlon C202 (E)
Rhododendron radians J.J.Sm.	Cultivated, USA, Washington, Federal Way, Rhododendron Species Botanical Garden, Accession No. 1997/ 063	Goetsch 070 (WTU)
Rhododendron rappardii Sleumer	Cultivated, UK, Scotland, Royal Botanic Garden Edinburgh, Accession No. 20090776	Argent *et al.* 39 (E)
Rhododendron rarilepidotum J.J.Sm.	Cultivated, UK, Scotland, Royal Botanic Garden Edinburgh, Accession No. 19881429	Conlon C126 (E)
Rhododendron rarum Schltr.	Cultivated, UK, Scotland, Royal Botanic Garden Edinburgh, Accession No. 19681098	Conlon C460 (E)
Rhododendron retivenium Sleumer #1	Cultivated, UK, Scotland, Royal Botanic Garden Edinburgh, Accession No. 19801288	Conlon C316 (E)
Rhododendron retivenium Sleumer #2	Cultivated, UK, Scotland, Royal Botanic Garden Edinburgh, Accession No. 19952750	Galloway 19 (E)
Rhododendron retusum (Blume) Benn.	Cultivated, USA, Washington, Federal Way, Rhododendron Species Botanical Garden, Accession No. 1979/027	Soza *et al.* 1930 (WTU)
Rhododendron rhodoleucum	Cultivated, UK, Scotland,	Conlon C221 (E)

Sleumer	Royal Botanic Garden Edinburgh, Accession No. 19682180	
Rhododendron robinsonii Ridl.	Cultivated, UK, Scotland, Royal Botanic Garden Edinburgh, Accession No. 19731358	Conlon C130 (E)
Rhododendron rousei Argent & Madulid	Cultivated, UK, Scotland, Royal Botanic Garden Edinburgh, Accession No. 19902330	Galloway 20 (E)
Rhododendron rubineiflorum Craven	Cultivated, UK, Scotland, Royal Botanic Garden Edinburgh, Accession No. 19962429	Conlon C327 (E)
Rhododendron rugosum H.Low ex Hook.f. var. *rugosum*	Cultivated, USA, Washington, Federal Way, Rhododendron Species Botanical Garden, Accession No. 1999/288	Goetsch 121 (WTU)
Rhododendron rushforthii Argent & D.F.Chamb.	Cultivated, USA, Washington, Federal Way, Rhododendron Species Botanical Garden, Accession No. 1997/087	N/A
Rhododendron ruttenii J.J.Sm.	Cultivated, UK, Scotland, Royal Botanic Garden Edinburgh, Accession No. 19880508	Conlon C197 (E)
Rhododendron santapaui Sastry & al.	Cultivated, UK, Scotland, Royal Botanic Garden Edinburgh, Accession No. 19830996	Conlon C206 (E)
Rhododendron saxifragoides J.J.Sm.	Cultivated, UK, Scotland, Royal Botanic Garden Edinburgh, Accession No. 19913932	Conlon C162 (E)
Rhododendron scortechinii King & Gamble	Cultivated, UK, Scotland, Royal Botanic Garden Edinburgh, Accession No. 20041584	Conlon C400 (E)
Rhododendron searleanum Sleumer	Cultivated, UK, Scotland, Royal Botanic Garden Edinburgh, Accession No. 19741176	Conlon C303 (E)

Rhododendron solitarium Sleumer	Cultivated, UK, Scotland, Royal Botanic Garden Edinburgh, Accession No. 19681395	Conlon C22 (E)
Rhododendron sp. nova	Cultivated, UK, Scotland, Royal Botanic Garden Edinburgh, Accession No. 20061814	Argent *s.n.* (E)
Rhododendron stapfianum Hemsl. ex Prain	Cultivated, UK, Scotland, Royal Botanic Garden Edinburgh, Accession No. 19821522	Conlon C203 (E)
Rhododendron stenophyllum Hook.f. ex Stapf subsp. *stenophyllum*	Cultivated, UK, Scotland, Royal Botanic Garden Edinburgh, Accession No. 19801190	Conlon C455 (E)
Rhododendron suaveolens Sleumer	Cultivated, UK, Scotland, Royal Botanic Garden Edinburgh, Accession No. 19762590	Conlon C268 (E)
Rhododendron superbum Sleumer	Cultivated, USA, Washington, Federal Way, Rhododendron Species Botanical Garden, Accession No. 1978/094	Goetsch 099 (WTU)
Rhododendron sutchuenense Franch.	Cultivated, USA, Washington, Federal Way, Rhododendron Species Botanical Garden, Accession No. 1965/348	N/A
Rhododendron taxifolium Merr.	Cultivated, USA, Washington, Federal Way, Rhododendron Species Botanical Garden, Accession No. 2013/241	Soza & Ramage 1954 (WTU)
Rhododendron tuba Sleumer	Cultivated, UK, Scotland, Royal Botanic Garden Edinburgh, Accession No. 19830538	Conlon C64 (E)
Rhododendron vaccinioides Hook.f.	Cultivated, USA, Washington, Federal Way, Rhododendron Species Botanical Garden, Accession No. 1999/308	N/A
Rhododendron vidalii Rolfe subsp. *vidalii*	Cultivated, UK, Scotland, Royal Botanic Garden Edinburgh, Accession No.	Argent *s.n.* (E)

	19972468	
Rhododendron vinicolor Sleumer	Cultivated, UK, Scotland, Royal Botanic Garden Edinburgh, Accession No. 19990504	Conlon C469 (E)
Rhododendron vitis-idaea Sleumer	Cultivated, UK, Scotland, Royal Botanic Garden Edinburgh, Accession No. 19722383	Conlon C183 (E)
Rhododendron wentianum Koord.	Cultivated, UK, Scotland, Royal Botanic Garden Edinburgh, Accession No. 19951950	Argent *s.n.* (E)
Rhododendron womersleyi Sleumer	Cultivated, USA, Washington, Federal Way, Rhododendron Species Botanical Garden, Accession No. 1999/738	Soza 1962 (WTU)
Rhododendron wrightianum Koord. var. *wrightianum*	Cultivated, USA, Washington, Federal Way, Rhododendron Species Botanical Garden, Accession No. 1994/375	N/A
Rhododendron yelliotii Warb.	Cultivated, UK, Scotland, Royal Botanic Garden Edinburgh, Accession No. 19861767	Conlon C97 (E)
Rhododendron zoelleri Warb	Cultivated, UK, Scotland, Royal Botanic Garden Edinburgh, Accession No. 19550409	Conlon C16 (E)
Rhododendron zollingeri J.J.Sm.	Cultivated, UK, Scotland, Royal Botanic Garden Edinburgh, Accession No. 19981653	Argent *s.n.* (E)

[1]Not applicable (N/A).

Table S2 Characteristics for each dataset used in Rhododendron sect. Schistanthe analyses.

Characters (for analyses)/Dataset	min4	min37	min74	min111
No. loci	120,904	36,655	20,029	6,575
Total bp (for RAxML)	11,093,231	3,377,539	1,834,035	599,067
Total SNPs (for Dsuite)	1,501,380	781,316	468,512	155,808
Unlinked SNPs (for SVDQuartets)	117,121	36,644	20,028	6,575

Methods S1 Library preparation

Samples with insufficient initial DNA concentrations were re-extracted as follows. We increased incubation of the sample in Qiagen P3 buffer to 30 min and omitted binding of the DNA to the DNeasy Mini spin column. Instead, we precipitated the cleared lysate from the Qia-shredder column using isopropanol, followed by a second precipitation using ethanol and sodium acetate. We then estimated DNA quantity using a Qubit® 2.0 Fluorometer (Invitrogen™, Life Technologies™, Carlsbad, CA, USA).

Low concentration DNAs were digested in multiple reactions, then combined and concentrated using Agencourt® AMPure XP (Beckman Coulter, Indianapolis, IN, USA). P1 adapter-ligated DNA was pooled and sheared using a Bioruptor® Pico (Diagenode Inc, Denville, NJ, USA) according to the manufacturer's protocol. Sheared DNA was size selected for 300- to 500-bp fragments by electrophoresis on 1% 1X TAE agarose. After RAD tag enrichment, DNA was size selected again for 300- to 500-bp fragments and verified by qPCR using the KAPA Universal Library Quantification Kit (Roche Sequencing, Pleasanton, CA, USA).

Methods S2 Data processing

We used a number of bioinformatic tools to further process the restriction-site associated DNA (RAD) data. To determine the optimal clustering threshold to use in *ipyrad*, we followed the method of Ilut *et al.* (2014). SEED (Bao *et al.*, 2011) was used to remove redundant reads from each sample's data. Custom Python scripts removed sequences with less than four reads. SlideSort v2 (Shimizu & Tsuda, 2010) found matching pairs of reads at distance thresholds from one to 20 under default settings. Transitive clusters were then generated using scripts kindly provided by D. Ilut (Cornell University, Ithaca, NY, USA) and visualized in R v4.0.3 (R Core Team, 2020) using ggplot2 (Wickham, 2016). The optimal clustering threshold was determined based on maximization of clusters with two haplotypes and minimization of clusters with one haplotype (Supporting Information Fig. **S1**).

Next, we filtered reads again using *ipyrad* with level two strictness for filtering adapters. We then conducted a reference-based assembly of loci within *ipyrad* under default settings, a clustering threshold of 0.91, as determined above, and a minimum depth of ten (Ilut *et al.*, 2014) for statistical base calling and majority-rule base calling. To determine the optimum values for maximum number of indels, maximum number of SNPs, and maximum shared heterozygosity allowed per locus, we followed the method of Nieto-Montes de Oca *et al.* (2017)

and visualized our results in R v4.0.3. Based on these results (Supporting Information Fig. **S2a-c**), we allowed a maximum of eight indels, 34 SNPs, and 0.3 shared heterozygosity per locus to obtain alignments for phylogenetic inference.

Methods S3 Phylogenetic analyses
First, we performed a maximum likelihood (ML) topology search in RAxML v8.2.11 (Stamatakis, 2014) using the concatenated alignment of all loci from *ipyrad* for each of the four datasets (Supporting Information Table **S2**). We specified *Kalmia* and *Kalmiopsis* as outgroups and ran a rapid bootstrap analysis and search for the best-scoring ML tree under the GTRCAT model, using the extended majority-rule consensus tree bootstopping criterion (Pattengale *et al.*, 2009).

Second, we reconstructed topologies for each dataset using the multispecies coalescent model in SVDQuartets (Chifman & Kubatko, 2014, 2015) on a concatenated alignment of one SNP sampled per locus from *ipyrad* (Supporting Information Table **S2**). For these analyses, we only included close outgroups from subg. *Rhododendron*. We conducted analyses in PAUP* v4.0a159 or 4.0a161 (Swofford, 2003) with 100 standard bootstrap replicates, evaluating all possible quartets, building trees using the Quartet FM (Reaz *et al.*, 2014) quartet assembly, using the multispecies coalescent tree model, and distributing ambiguities. We then summarized topologies using Consense v3.6.6 (Felsenstein, 2005) on the CIPRES Science Gateway v3.3 (Miller *et al.*, 2010) to compute a majority rule extended consensus tree.

To assess our two phylogenetic reconstruction methods on each of the four datasets, we used both bootstrap (bs) support and Quartet Sampling v1.3.1 (Pease *et al.*, 2018). First, we calculated the percent of bootstrap values for each topology that had support greater than or equal to 70% to find the highest supported topology from each reconstruction method. We then used Quartet Sampling on each of the four topologies resulting from the four datasets for each reconstruction method. We used the concatenated alignment of loci for each dataset with its respective topologies to obtain enough information for Quartet Informativeness (QI). We conducted Quartet Sampling with 500 replicates under default settings using RAxML-NG v0.9.0 (Kozlov *et al.*, 2019) as the engine. We visualized bootstrap and Quartet Sampling scores on topologies in R v4.0.3 using treeio v1.12.0 (Wang *et al.*, 2020) and ggtree v2.2.4 (Yu, 2020).

Methods S4 Introgression analyses
Reconstructed species relationships within clades varied among datasets and among phylogenetic reconstruction methods. Therefore, we assessed whether admixture could be a factor for incongruences using the *D*-statistic (ABBA-BABA test) (Green *et al.*, 2010; Durand *et al.*, 2011) as implemented in Dsuite v0.3r21 (Malinsky *et al.*, 2020). We used Dtrios on all SNPs resulting from *ipyrad* to calculate the *D*-statistic using three methods under default settings: (1) computing the *D*-statistic for all possible trios of species, without assuming any a priori knowledge of relationships, then using the lowest *D*-statistic (D_{min}) for each trio as a conservative estimate, (2) inferring species relationships using the frequency of BBAA patterns, then calculating discordant site patterns with the *D*-statistics (D_{BBAA}), and (3) using the topology

from our reconstruction methods, with the most support for clades and their relationships, as a guide for relationships in computing the *D*-statistic (D_{tree}) (Malinsky *et al.*, 2018, 2020).

Methods S5 Molecular dating

We used fossil calibration points for *Erica*, *Kalmia*, and *Rhododendron* and a maximum age for the root: 5.33, 15.97, 56, and 89.8 mya, respectively (Collinson & Crane, 1978; Van Der Burgh, 1987; Nixon & Crepet, 1993; Mai, 2001). We first conducted a priming analysis to determine the best optimization parameters, followed by a random subsample and replicate cross validation to determine the optimum smoothing value. We then conducted a thorough analysis in treePL on the bootstrap replicates generated above under default settings, but with a smoothing value of 0.1, gradient based optimizer of 2, auto-differentiation based optimizer of 2, and auto-differentiation cross validation based optimizer of 1. We summarized the dated bootstrap replicates with TreeAnnotator v2.6.0 (Bouckaert *et al.*, 2019) to obtain confidence intervals. However, the confidence intervals were very narrow, so we just report the mean ages in the chronogram.

Methods S6 Morphometric analyses

For the front of the corolla, most outlines were generated by reflecting the half of the corolla that was in best shape or easiest to extract. For the side of the corolla, we attempted outlines either closed (i.e., with a line connecting petal lobes to form an unbroken outline) or open at the mouth. After comparing both, we determined that open outlines preserved the whole side better for some species with reflexed petals. For grouping taxa in morphospaces by corolla color, color was assigned to taxa based on the three categories used by Stevens (1976) to describe floral types in sect. *Schistanthe*: red, yellow, and white. In cases where a taxon was polymorphic in color, we used the corolla color represented in our sampled taxon.

References

Bao E, Jiang T, Kaloshian I, Girke T. **2011**. SEED: efficient clustering of next-generation sequences. *Bioinformatics* **27**: 2502–2509.

Bouckaert R, Vaughan TG, Barido-Sottani J, Duchêne S, Fourment M, Gavryushkina A, Heled J, Jones G, Kühnert D, De Maio N, *et al.* 2019. BEAST 2.5: an advanced software platform for Bayesian evolutionary analysis. *PLOS Computational Biology* **15**: e1006650.

Chifman J, Kubatko L. **2014**. Quartet inference from SNP data under the coalescent model. *Bioinformatics* **30**: 3317–3324.

Chifman J, Kubatko L. **2015**. Identifiability of the unrooted species tree topology under the coalescent model with time-reversible substitution processes, site-specific rate variation, and invariable sites. *Journal of Theoretical Biology* **374**: 35–47.

Collinson ME, Crane PR. **1978**. Rhododendron seeds from the Palaeocene of southern England. *Botanical Journal of the Linnean Society* **76**: 195–205.

Durand EY, Patterson N, Reich D, Slatkin M. **2011**. Testing for ancient admixture between closely related populations. *Molecular Biology and Evolution* **28**: 2239–2252.

Felsenstein J. **2005**. *PHYLIP (Phylogeny Inference Package)*. Seattle, WA, USA: University of Washington.

Green RE, Krause J, Briggs AW, Maricic T, Stenzel U, Kircher M, Patterson N, Li H, Zhai W, Fritz MH-Y, *et al.* **2010**. A draft sequence of the Neandertal genome. *Science* **328**: 710–722.

Ilut DC, Nydam ML, Hare MP. **2014**. Defining loci in restriction-based reduced representation genomic data from nonmodel species: sources of bias and diagnostics for optimal clustering. *BioMed Research International* **2014**: 675158.

Kozlov AM, Darriba D, Flouri T, Morel B, Stamatakis A. **2019**. RAxML-NG: a fast, scalable and user-friendly tool for maximum likelihood phylogenetic inference. *Bioinformatics* **35**: 4453–4455.

Mai HD. **2001**. Die mittelmiozänen und obermiozänen Floren aus der Meuroer und Raunoer Folge in der Lausitz. Teil II: Dicotyledonen. *Palaeontographica Abteilung B* **257**: 35–174.

Malinsky M, Matschiner M, Svardal H. **2020**. Dsuite - fast *D*-statistics and related admixture evidence from VCF files. *Molecular Ecology Resources* **00**: 1–12.

Malinsky M, Svardal H, Tyers AM, Miska EA, Genner MJ, Turner GF, Durbin R. **2018**. Whole-genome sequences of Malawi cichlids reveal multiple radiations interconnected by gene flow. *Nature Ecology & Evolution* **2**: 1940–1955.

Miller MA, Pfeiffer W, Schwartz T. **2010**. Creating the CIPRES Science Gateway for inference of large phylogenetic trees. In: Proceedings of the Gateway Computing Environments Workshop (GCE), 2010. New Orleans, LA: Institute of Electrical and Electronics Engineers, 1–8.

Nieto-Montes de Oca A, Barley AJ, Meza-Lázaro RN, García-Vázquez UO, Zamora-Abrego JG, Thomson RC, Leaché AD. **2017**. Phylogenomics and species delimitation in the knob-scaled lizards of the genus *Xenosaurus* (Squamata: Xenosauridae) using ddRADseq data reveal a substantial underestimation of diversity. *Molecular Phylogenetics and Evolution* **106**: 241–253.

Nixon KC, Crepet WL. **1993**. Late Cretaceous fossil flowers of Ericalean affinity. *American Journal of Botany* **80**: 616–623.

Pattengale ND, Alipour M, Bininda-Emonds ORP, Moret BME, Stamatakis A. **2009**. How many bootstrap replicates are necessary? In: Batzoglou S, ed. Research in Computational Molecular Biology: 13th Annual International Conference, RECOMB 2009, Tucson, AZ, USA, May 18-21, 2009. Proceedings. Berlin, Heidelberg: Springer Berlin Heidelberg, 184–200.

Pease JB, Brown JW, Walker JF, Hinchliff CE, Smith SA. **2018**. Quartet Sampling distinguishes lack of support from conflicting support in the green plant tree of life. *American Journal of Botany* **105**: 385–403.

R Core Team. **2020**. *R: A language and environment for statistical computing.* Vienna, Austria: R Foundation for Statistical Computing.

Reaz R, Bayzid MS, Rahman MS. **2014**. Accurate phylogenetic tree reconstruction from quartets: a heuristic approach. *PLoS ONE* **9**: e104008.

Shimizu K, Tsuda K. **2010**. SlideSort: all pairs similarity search for short reads. *Bioinformatics* **27**: 464–470.

Stamatakis A. **2014**. RAxML version 8: a tool for phylogenetic analysis and post-analysis of large phylogenies. *Bioinformatics* **30**: 1312–1313.

Stevens PF. **1976**. The altitudinal and geographical distributions of flower types in *Rhododendron* section *Vireya*, especially in the Papuasian species, and their significance. *Botanical Journal of the Linnean Society* **73**: 1–33.

Swofford DL. **2003**. *PAUP*. Phylogenetic Analysis Using Parsimony (*and Other Methods)*. Sunderland, MA, USA: Sinauer Associates.

Van Der Burgh J. **1987**. Miocene floras in the lower Rhenish Basin and their ecological interpretation. *Review of Palaeobotany and Palynology* **52**: 299–366.

Wang L-G, Lam TT-Y, Xu S, Dai Z, Zhou L, Feng T, Guo P, Dunn CW, Jones BR, Bradley T, *et al.* **2020**. treeio: an R package for phylogenetic tree input and output with richly annotated and associated data. *Molecular Biology and Evolution* **37**: 599–603.

Wickham H. **2016**. *ggplot2: Elegant Graphics for Data Analysis*. New York, NY, USA: Springer-Verlag.

Yu G. **2020**. Using ggtree to visualize data on tree-like structures. *Current Protocols in Bioinformatics* **69**: e96.
